# Supplementary material for: The impact of new urbanization on “quantity increase and quality improvement” of urban green innovation
Source: PLoS One. 2026 May 19;21(5):e0329052. doi: 10.1371/journal.pone.0329052 (PMC13186382; doi:10.1371/journal.pone.0329052)
Supplement: Attachment — Submitted filename: Response to Reviewers.docx [file pone.0329052.s001.docx]

# Response to Reviewers’ Comments

# (Manuscript Number: PONE-D-25-37471)

# Dear editor and reviewers,

Thank you for your letter and for the reviewers’ valuable and helpful comments on our manuscript entitled “The impact of new urbanization on quantity increase and quality improvement of green innovation: based on multivariate moderating effect models” (ID: PONE-D-25-37471). We have carefully read all comments and have made corresponding corrections. We hope that our revisions have significantly improved the quality of the paper. The modified content has been marked to highlight the changes. The responses to the reviewers’ comments are as follows.

***Response to Reviewer #1 Comments***

**Reviewer #1:**

This article explores the urban development program focusing on green innovations, which is noteworthy in itself. This article investigates the urban development program concerning green innovations, which is intriguing in its own way. This article analyzes the urban development initiative aimed at green innovations, which is captivating on its own. The analytical approach is also grounded in statistical models;

The methodology of analysis is likewise rooted in statistical models;

which can be accomplished using historical data.

which can be executed based on historical data.

which can be achieved on the foundations of historical data.

**Response:**

We sincerely appreciate the reviewer's thoughtful evaluation and insightful comments on our manuscript, particularly the recognition of the value in our analysis of the urban development program concerning green innovations. This validates the significance of our research focus, strengthens our confidence in exploring the interdisciplinary nexus between sustainability and urban planning, and motivates us to refine our methodologies to enhance both academic rigor and practical applicability.

**Comment 1:**

Regarding the significant volume of calculations, some aspects must be elucidated:

Where are the analyzed data sourced from?

**Response:**

We sincerely appreciate the reviewer for emphasizing the critical need to clarify the data sources on which our analysis is based. We fully acknowledge that declaring the source of data is essential for analyzing data and validating results. However, we regret that this aspect was not adequately addressed in the original submission. To clarify the specific sources of the data more explicitly, we have added a new section titled "3.3 Data sources" in Part 3 (3. Research design) of the paper. In this section, we have provided detailed information on the data sources for each variable, including specific databases, government reports, and public datasets. The specific details are outlined below:

*This paper utilizes panel data from 284 cities spanning the period from 2011 to 2022. The data for GIN and GNS are from the Chinese Research Data Services Platform (CNRDS), NEU encompasses multiple second-level indicators, and the data for these indicators mainly originate from the China Urban Statistical Yearbook, statistical yearbooks of various regions, the EPS database and the China Energy Statistical Yearbook. The data for control variables FDI, TEL, OOW, HCL, RFV and SCL are from the China Urban Statistical Yearbook, and the data for INT is from the EPS database. The data for DIF is sourced from the Digital Financial Inclusion Index published by Peking University. The data for moderating variables GTS, INT, AGDP are from the China Urban Statistical Yearbook, and the data for ENC is from the China Energy Statistical Yearbook. The data for GES is from the zldatas database.*

***Response to Reviewer #2 Comments***

**Reviewer #2:**

The paper addresses a highly relevant and timely topic, especially in the context of China's national strategy for sustainable development. The methodology employed is robust, including various econometric models and comprehensive checks for robustness and endogeneity. The findings offer significant policy implications, making a valuable contribution to the literature.

**Response:**

We sincerely appreciate the reviewer's thorough review and insightful comments provided on our manuscript. The comments reassure us that our analytical framework resonates with scholarly standards in applied economics. The comments also motivate us to refine these models further in future research.

**Comment 1:**

1. Even though the paper uses green patent applications (GIN) and green invention patent applications (GNS) as measures for the "quantity increase" and "quality improvement" of GI respectively , it would be beneficial to briefly acknowledge the potential limitations of relying solely on patent data as a proxy for all green innovation (e.g., not all innovations are patented, varying implementation rates, or challenges in capturing the full scope of GI).

**Response:**

We thank the reviewer for the valuable comments and we completely agree that relying solely on patent data as a proxy for all green innovation has potential limitations. As the reviewer mentioned, not all innovations are patented, and there are varying implementation rates among different innovations. Moreover, it is indeed challenging to capture the full scope of green innovation using only patent data. We have made modifications to the paper according to the reviewer’s suggestion. In the relevant section where we have introduced the use of green patent applications and green invention patent applications as measures, we have added a discussion on these limitations. We have elaborated on how these limitations may affect the accuracy and comprehensiveness of our research results, and also discussed possible ways to mitigate these limitations in future research. We have added the content of “6.3. Limitations and future research” in the last section “6. Discussion” of the paper. We believe that adding this discussion will make our research more rigorous and comprehensive and enhance the credibility of our findings. Specifically, the following content has been added:

*It should be pointed out that while we use green patent applications and green invention patent applications as measures for the "quantity increase" of GI and "quality improvement" of GI, respectively, relying solely on patent data as a proxy for all GI has several potential limitations. For example, the level of GI may have been underestimated; not all innovations are patented, the implementation rates of patented GI can vary significantly, and not all green patents can be applied in practice. It is challenging to capture the full scope of GI using patent data. In order to further overcome these limitations, patent data can be combined with other data in future research on GI, and environmental performance indicators, energy efficiency, etc., can be included in the analysis of GI to more comprehensively cover the scope of GI.*

**Comment 2:**

2. The current "Discussion" section (Section 6) functions primarily as a "Summary" and "Recommendations" . To maximize the paper's impact, a dedicated "Discussion" subsection could be expanded to:

o More explicitly contextualize the findings within the broader literature, highlighting how they extend, confirm, or diverge from previous research.

o Elaborate on the theoretical implications of the findings.

o Discuss potential limitations of the study (e.g., generalizability beyond China, reliance on specific data proxies) that are not already covered by the robustness checks.

**Response:**

We would like to thank the reviewer for the thoughtful and constructive suggestions. We have carefully considered each of the comments and made significant revisions. We indeed should further expand the content of the "Discussion" section (Section 6). We have revised the 'Discussion' section of the paper to better align our findings with existing literature. The following is a detailed description of our modifications:

(1) We have integrated the summarized content into the discussion section (Section 6) by comparing and discussing existing literature to clearly demonstrate the relationship between our research and previous studies. The following specific content has been added to the "6.1. Summary" section:

*Our research provides detailed empirical evidence through empirical analysis using urban panel data. It not only confirms the viewpoints of previous studies but also expands upon them by providing concrete evidence. DIF significantly amplifies the positive impact of NEU on GI, which is a new perspective and expands the application of DIF in the field of GI. This multi-factor interaction analysis provides a more comprehensive understanding of NEU and GI and extends previous single-factor or limited-factor studies. GES plays a crucial role in enhancing the impact of NEU on GIN and GNS, which confirms the overall positive impact of government support on innovation and expands research by exploring the specific role of GES in the relationship between NEU and GI.*

(2) We have expanded the content of theoretical implications in the Discussion section (6. Discussion) by adding a new subsection "6.2. Theoretical and policy implications" to provide a more in-depth analysis and elaborate on the theoretical contribution of this study. The following specific content has been added:

*Our research focuses on the impact of NEU on GI, challenging the traditional view that urbanization mainly focuses on economic growth and resource utilization. NEU emphasizes green development and ecological benefits, and has a more obvious promoting effect on GI. It is consistent with the viewpoint of the new urban development theory, providing a new theoretical perspective on how urbanization can promote GI and enriching existing theories on the relationship between urbanization and innovation. DIF amplifying the impact of NEU on GI indicates the need to further strengthen attention to the theory of enhancing financial inclusiveness and industrial integration, which opens up a new path for the integration of digital technology innovation and green development concepts into urban management and planning. The study of the interaction of multiple factors emphasizes the importance of multi-factor interaction in innovation theory, which provides a more comprehensive understanding of innovation theory and helps to formulate more effective policies to promote GI.*

(3) We have also added new content in section “6.3. Limitations and future research", the potential limitations are discussed in detail, and directions for future research to address these issues are proposed. The following specific content has been added:

*China has a distinctive social system and urbanization process, and this study is mainly based on the analysis of China's urban panel data, which will inevitably affect the applicability of the research conclusions to other countries. Therefore, future research can further expand the scope of the study, analyze the relationship between urbanization and GI in other countries, and compare it with China to test the boundary conditions of our research results. In addition, although we have employed various methods in the paper to test the robustness and endogeneity of the analysis results, in order to alleviate concerns about reliability on specific data proxies, data limitations still exist. Future research can adopt various research methods, such as incorporating qualitative research methods or adopting quasi-experimental research methods, to provide more causal evidence for the relationship between NEU and GI.*

**Comment 3:**

3. While the English is generally clear, there are a few instances of phrasing that could be slightly refined for improved conciseness and flow (e.g., "amplify the dividends of new urbanization and exert the GI effect"; "the regression results are resilient" ; "transformation from quantity to quality" ). A final proofread by a native English speaker could further polish the manuscript.

**Response:**

We would like to apologize to the reviewer for the language used in our manuscript. Perhaps due to repetitive content modifications, there are some language issues. We have invited professionals in the field of English language to examine the language used in our paper, discussed each specific example mentioned, and provided a revised version. We really hope to have a substantial improvement in language proficiency. The modifications made to some phrases are presented as follows, some modifications to other statements and phrases have been marked in the paper.

**Original phrase:** "amplify the dividends of new urbanization and exert the GI effect"

**Revised phrase:** “unleash the development dividends brought by NEU and fully leverage the positive effects of GI.”

**Original phrase:** "the regression results are resilient"

**Revised phrase:** "the regression results exhibit robustness"

**Original phrase:** NEU in China is in the development stage of "quantity is stronger than quality" and "transformation from quantity to quality".

**Revised phrase:** NEU in China is in a transitional phase from "quantity-based growth" to "quality-oriented enhancement".

**Original phrase:** “NEU is playing an increasingly significant role in GI through different ways, such as talent pooling, consumption upgrading, and development mode.”

**Revised phrase:** “At the critical stage of current social development, NEU, as a systematic and comprehensive strategy, is exerting increasingly significant and far-reaching effects on GI from different dimensions, effectively promoting the rapid development and efficient application of GI in various fields.”

**Original phrase:** “the regression coefficients are unchanged, which confirms the reliability of the benchmark regression”

**Revised phrase:** “there is no obvious alterations in the positive/negative orientations or quantitative value of the coefficient, which verifies the validation for the results”

**Original phrase:** “high informatization cities can more efficiently match digital financial resources with green technology needs, reduce transaction costs, capital, technology, and talent can flow more efficiently to the field of GI, which strengthening the connection between NEU and GI.”

**Revised phrase:** “in high informatization cities, with their advanced digital infrastructure and efficient information dissemination mechanisms, it is possible to achieve more accurate and rapid matching between digital financial resources and green technology needs. This precise matching effectively reduces transaction costs, thereby promoting key production factors such as capital, technology, and talent to cluster towards the field of GI with higher efficiency, ultimately significantly strengthening the intrinsic influence between NEU and GI.”

**Original phrase:** “amplifies the dividends of NEU and exert strong "quantity increase" and "quality improvement" effects on GI.”

**Revised phrase:** “fully unleash the development dividends brought by NEU and exert strong "quantity increase" and "quality improvement" effects of GI”

***Response to Reviewer #3 Comments***

**Reviewer #3:**

This paper analyzes whether China’s new urbanization (NEU) drives both the quantity and the quality of green innovation. Manuscript satisfactory.

**Response:**

We sincerely thank the reviewer's thoughtful evaluation and insightful comments on our manuscript. The reviewer's comments boost our confidence and enthusiasm for this research. We'll use this motivation to revise the paper further and try our best to improve its quality.

**Comment 1:**

However, standardize acronym use, by defining NEU, GI, DIF, GTS, INT, AGDP, ENC, GES once, then use consistently.

**Response:**

We sincerely appreciate the reviewer’s attention to this critical detail of consistent use of acronyms in our manuscript. We fully agree that standardized terminology can improve clarity and readability. As suggested, we have now defined all acronyms when they first appear in the text, marked their abbreviations in parentheses, and ensured their consistent use throughout the manuscript. We have also checked the entire document to eliminate any inconsistent or inexplicable abbreviations. The definition of each acronym is as follows:

GI: green innovation

NEU: new urbanization

GIN: “quantity increase” of GI

GNS: "quality improvement" of GI

CV: control variables

FDI: foreign direct investment

TEL: technology investment level

OOW: opening to the outside

HCL: human capital level

INT: the Internet penetration rate

RFV: the per capita volume of road freight transportation

SCL: the level of social consumption

DIF: Digital Inclusive Finance

GTS: government technology support

INT: informatization

AGDP: per capita GDP

ENC: energy consumption

NEM: new-energy model cities policy

CIT: Citizenship of the rural transferring population

NGIN: the alternative variable for GIN, measured by the total number of green patents granted per 10,000 people

NGNS: the alternative variable for GNS, measured by the total number of green invention patents granted per 10,000 people

NLV: night lighting value

GES: government green support

The specific modifications have been marked in the paper.
