# Supplementary material for: The impact of new urbanization on “quantity increase and quality improvement” of urban green innovation
Source: PLoS One. 2026 May 19;21(5):e0329052. doi: 10.1371/journal.pone.0329052 (PMC13186382; doi:10.1371/journal.pone.0329052)
Supplement: Attachment — Submitted filename: Response_to_Reviewers_auresp_2.docx [file pone.0329052.s002.docx]

# Response to Reviewers’ Comments

# (Manuscript Number: PONE-D-25-37471R1)

# Dear Reviewers,

We would like to express our heartfelt appreciation to the reviewers for your meticulous evaluation and insightful technical suggestions which have significantly strengthened our manuscript entitled “The impact of new urbanization on quantity increase and quality improvement of green innovation: based on multivariate moderating effect models” (ID: PONE-D-25-37471R1). Below is our point-by-point response to the comments:

***Response to Reviewer #1 Comments***

**Reviewer #1:**

After reviewing your manuscript, I have some technical suggestions to improve its quality, ensuring the availability of the necessary data:

It is recommended to use exploratory or confirmatory factor analysis, and possibly both, on the data. Additionally, principal component analysis can be beneficial for understanding the relationships among independent variables and the degree of their interconnections, leading to deeper and more advanced conclusions. Furthermore, utilizing residual plots against fitted values or error-related indices for evaluating regression results, especially in equations with high R² values, can enhance the quality of the manuscript's conclusions.

I am concerned about the lack of understanding of the relationships among the independent variables in this manuscript. Using Cronbach's alpha to demonstrate these relationships, along with presenting a fitted distribution curve for the data, seems essential for better understanding the nature of the data.

**Response:**

We sincerely thank the reviewer for the constructive feedback and detailed technical suggestions. Your technical suggestions are excellent and will undoubtedly significantly improve the quality of our work. We have carefully considered all your points and have developed a detailed revision plan to address them comprehensively. The key revisions we undertake are as follows:

1. This paper employs Exploratory Factor Analysis (EFA) and principal component analysis (PCA) to further examine the relationships and intercorrelations among the independent variable data. First, Cronbach’s alpha values are calculated for each indicator to assess data reliability. Then, the Kaiser-Meyer-Olkin (KMO) measure and Bartlett’s test of sphericity are conducted to evaluate the partial correlations among variables and the correlation matrix. All statistical measures meet the required criteria for the tests. Furthermore, as a part of the robustness checks, this study recalculated the explanatory variable NEU by replacing the composite score derived from the entropy weight method with a factor composite score obtained through Exploratory Factor Analysis (EFA). This approach is implemented in section "iv) Replacing independent variable" under "4.2 Robustness test" to verify the stability of the regression results.

Specific tests and indicators have been added to the section "ii) Independent variable" under "3.1. Indicator design" of the paper. The details are as follows:

*In order to verify that there is a good reliability and correlation between the third-level indicators of NEU. Firstly, the reliability of the data is checked. The reliability of the data is measured by the Cronbach's alpha coefficient, and the Cronbach's alpha value is calculated for each of the three-level metrics as well as for all the metrics, and the results are obtained as shown in Table 2. The Cronbach's alpha value of each metric is greater than 0.7, and the Cronbach's alpha value of the overall reliability of the data is also greater than 0.7, which shows that the data satisfy the reliability requirements. Secondly, KMO and Bartlett's spherical test are utilized to assess the partial correlation and correlation matrix between the variables, as shown in Table 3. The result of the test is overall value of KMO is 0.7792>0.6, Bartlett of sphericity passes the test at the 0.01 level, indicating a good correlation and validity between the variables.*

***Table 2 Cronbach's Alpha of Indicators of NEU***

| *Variable* | *Cronbach's alpha* | *Variable* | *Cronbach's alpha* | *Code* | *Cronbach's alpha* |
| --- | --- | --- | --- | --- | --- |
| *X1* | *0.816* | *X12* | *0.8293* | *NEU* | *0.838* |
| *X2* | *0.8332* | *X13* | *0.8302* |  |  |
| *X3* | *0.828* | *X14* | *0.8181* |  |  |
| *X4* | *0.8266* | *X15* | *0.8319* |  |  |
| *X5* | *0.8428* | *X16* | *0.8339* |  |  |
| *X6* | *0.8253* | *X17* | *0.8314* |  |  |
| *X7* | *0.8395* | *X18* | *0.8357* |  |  |
| *X8* | *0.8334* | *X19* | *0.8266* |  |  |
| *X9* | *0.8377* | *X20* | *0.8467* |  |  |
| *X10* | *0.819* | *X21* | *0.8406* |  |  |
| *X11* | *0.8319* | *X22* | *0.8312* |  |  |

***Table 3 KMO and Bartlett Test of Sphericity Results***

| *Variable* | *KMO* | *Variable* | *KMO* | *Variable* | *KMO* | *Bartlett test of sphericity* | |
| --- | --- | --- | --- | --- | --- | --- | --- |
| *X1* | *0.7854* | *X12* | *0.8509* | *Overall* | *0.7792* | *Chi-square* | *33184.396**** |
| *X2* | *0.5740* | *X13* | *0.8951* |  |  |  |  |
| *X3* | *0.8821* | *X14* | *0.8408* |  |  |  |  |
| *X4* | *0.7905* | *X15* | *0.8423* |  |  |  |  |
| *X5* | *0.6430* | *X16* | *0.8999* |  |  |  |  |
| *X6* | *0.8571* | *X17* | *0.7079* |  |  |  |  |
| *X7* | *0.6819* | *X18* | *0.8400* |  |  |  |  |
| *X8* | *0.6446* | *X19* | *0.7768* |  |  |  |  |
| *X9* | *0.4807* | *X20* | *0.6384* |  |  |  |  |
| *X10* | *0.8891* | *X21* | *0.6939* |  |  |  |  |
| *X11* | *0.8845* | *X22* | *0.7233* |  |  |  |  |

2. This study utilizes the relationship between residual plots and fitted values to evaluate the regression results, ensuring that the model meets the assumptions of normality, homoscedasticity, and independence. According to statistical theory, regression residuals should approximately follow a normal distribution with a mean of zero. If the residual distribution exhibits deviations from the zero line, curved patterns, or funnel shapes, it may indicate model misspecification or omission of important variables, potentially leading to biased regression results. Therefore, residual evaluation is conducted on the benchmark regression results of the impact of NEU on the "quantity increase" of GI (GIN) and the "quality improvement" of GI (GNS). The following analysis has been added to the "4.1 Benchmark Regression" section of the paper.

*To further verify that the explanatory variable NEU affects GIN and GNS model meets the requirements of, normality, chi-square and independence, this paper evaluates the plots of fitted distributions of the residuals of the regressions of NEU on GIN and GNS. First, the fitted values and residuals are calculated for the regression results of NEU affecting GIN, and the plots of residual fitted values are plotted as shown in Figure 1. The scatter points are uniformly distributed on both sides of the reference line at y = 0. There is no systematic bias where all points lie above or below the reference line, indicating that the model is well unbiased and does not consistently overestimate or underestimate the observations. The scatter cloud is randomly distributed and does not form U-shaped, inverted U-shaped, or curved banding patterns, indicating that the main linear relationships of the data have been captured in the model and no important nonlinearities have been missed. The vertical range of fluctuations in the residuals is generally consistent over the vast majority of the range of fitted values (X-axis), with no curvilinear trends, funnel shapes, or other systematic patterns, a good indication of satisfying the assumption of homoscedasticity. Plotting the normal probability of the residuals (Q-Q plots) meets the linear regression model's requirement for normality of the residuals. Next, the fitted values and residuals are calculated for the regression results of NEU affecting GNS, and the residual fitted values are plotted, as shown in Figure 2. The scatter fluctuates roughly around and above the horizontal axis (residuals are 0) and there is no obvious pattern in the distribution of data points, indicating that the model is not systematically biased, i.e., it does not consistently overestimate or underestimate the actual values. The absence of hidden nonlinear patterns in the residuals indicates that the model has successfully captured the main linear relationships in the data. The range of fluctuations in the residuals is relatively stable over the entire range of fitted values, and the scatter cloud does not show a clear "funnel shape". Plotting the normal probability of the residuals (Q-Q plot) meets the requirement of residual normality for the linear regression model.*

**Figure 1 NEU Affects GIN's Residual Error vs. Fitted Value Graph**

**Figure 2 NEU Affects GNS's Residual Error vs. Fitted Value Graph**

3. The original data used in this study have been uploaded to a public data platform, with detailed information provided in the "Data Availability Statement" section of the paper. Additionally, in order to further enhance data transparency and reproducibility, we have supplemented a file containing the raw data for the three-level indicators of the explanatory variable (New Urbanization, NEU) to the designated data repository.

The specific statement in the "Data Availability Statement" section of the paper is as follows:

DOI: 10.5281/zenodo.17547697

Cite as: Fang, L. (2025). The dataset for the paper titled "The impact of new urban-ization on quantity increase and quality improvement of green innovation: Based on multivariate moderating effect models" [Data set]. Zenodo. https://zenodo.org/records/17547697

***Response to Reviewer #2 Comments***

**Reviewer #2:**

This revised manuscript addresses a highly relevant and timely topic concerning the relationship between New Urbanization (NEU) and Green Innovation (GI) in the context of China's national development strategy. The study uses empirical analysis based on panel data from 284 cities spanning 2011 to 2022. The methodology appears strong, incorporating various econometric models, including fixed effects, robustness checks (removing special city/year samples, replacing variables), and instrumental variable methods (LAG_NEU and NLV) to address endogeneity concerns. The primary findings that NEU positively contributes to both the quantity increase (GIN) and quality improvement (GNS) of GI, and that Digital Inclusive Finance (DIF) significantly amplifies this impact offer significant policy implications and make a valuable contribution to the literature. Importantly, the authors are commended for making substantial revisions in response to the previous reviewers' comments, particularly regarding data transparency, discussion depth, and linguistic clarity. These revisions have significantly improved the quality and rigor of the manuscript.

**Response:**

Thank you very much for your positive and encouraging feedback on our revised manuscript. We are truly honored by your recognition of the study's relevance, methodological rigor, and substantive contributions. Your acknowledgment of the revisions made—particularly regarding data transparency, discussion depth, and linguistic clarity—is greatly appreciated and motivates us to continue striving for excellence in our research. We are glad that you find the empirical approach sound and the policy implications valuable. Your supportive comments reinforce our confidence in further exploring this important topic and pursuing rigorous scientific inquiry. Should any further refinements or clarifications be needed, we are more than willing to address them promptly.
