# Supplementary material for: The impact of new urbanization on “quantity increase and quality improvement” of urban green innovation
Source: PLoS One. 2026 May 19;21(5):e0329052. doi: 10.1371/journal.pone.0329052 (PMC13186382; doi:10.1371/journal.pone.0329052)
Supplement: Attachment — Submitted filename: PONE-D-25-37471_R2.docx [file pone.0329052.s003.docx]

Dear Editor,

I am writing to provide my review of the manuscript entitled “The Impact of New Urbanization on the Quantity and Quality of Green Innovation: Evidence from Multivariate Moderating Effect Models.” This paper examines whether new urbanization in China promotes both the quantity and the quality of green innovation using panel data from 284 cities during 2011–2022. The findings indicate that new urbanization significantly enhances green innovation, with digital inclusive finance and government-related factors further strengthening this positive effect.

To further enhance the clarity and impact of the research findings, I would like to offer the following suggestions:

1. Title

- Consider adopting a more concise and conceptually integrated title.

2. Abstract

- Add information on model types, sample size, and the direction and magnitude of key coefficients.
- Reduce repetition and tighten the wording.

3. Introduction

- Include a dedicated research-gap paragraph explicitly stating what previous studies have overlooked and why distinguishing between innovation quantity and quality is important.
- Strengthen the theoretical framing (e.g., innovation economics, environmental regulation theory, agglomeration theory, and digital finance theory).
- Reduce policy-oriented narrative and increase analytical synthesis of prior literature.

4. Research Hypotheses

- Explicitly link the hypotheses to established theoretical models.
- Provide a conceptual framework figure illustrating the causal pathways.
- Clarify whether the moderating effects are complementary, substitutive, or threshold-based.

5. Research Design

- Report VIF statistics and correlation diagnostics.
- Provide clearer transparency regarding data preprocessing procedures.

6. Empirical Analysis

- Interpret coefficients in terms of real-world economic magnitude.
- Consider applying spatial econometric or dynamic panel models.

7. Further Analysis

- Provide theoretical interpretation of the mechanisms, rather than relying solely on statistical significance.

8. Discussion

- Deepen the theoretical interpretation of the findings.
- Compare the results with international studies on urbanization and innovation.
- Add a clear subsection on limitations and directions for future research.

9. References

- Ensure consistency in DOI formatting.
- Confirm that all cited references appear in the reference list and that all listed references are cited in the text.
- Prioritize the most recent and relevant literature-preferably published between 2017 and 2026-to maintain the currency of the review. Where appropriate, removing outdated methodological references may further strengthen the manuscript.

Overall, I believe the manuscript has the potential to meet the standards required for publication and offers meaningful contributions to the field, provided that the revisions outlined above are carefully addressed.
