# Supplementary material for: The impact of new urbanization on “quantity increase and quality improvement” of urban green innovation
Source: PLoS One. 2026 May 19;21(5):e0329052. doi: 10.1371/journal.pone.0329052 (PMC13186382; doi:10.1371/journal.pone.0329052)
Supplement: Attachment — Submitted filename: GI.Rp2..docx [file pone.0329052.s004.docx]

**Review report (date: 26.2.2026)**

The Impact of new urbanization on quantity increase and quality improvement of green innovation: Based on multivariate moderating effect models, Manuscript Number: PONE-D-25-37471R2

**General issues** (taking into account seven guiding principles): Since I have viewed both the original and revised paper.

**A1**. The research is the original results of the study. The paper explores the linkage between new urbanisation and green innovation practices in Chinese cities, hence, presenting an example that can be used in policy and research in other nations and in filling the research gap in the field.

**A2.** There has been no publication of the results obtained. The manuscript utilises a micro-panel dataset spanning 2011 to 2022 (11 years) that makes the authors depict the time-related tendency in green innovation in the context of new urbanisation.

**A3**. Experiments, statistics, and other discussions are done to a high technical standard, and they are described in adequate detail. Basic pre-tests have been met, and the obtained estimates are sound, which makes it easy to replicate other researchers.

**A4**. The conclusions (summary) are made in a suitable way and are backed up by data. The conclusions are made logically out of the key findings and presented in a clear, scientific and concrete way.

**A5.** The article is written in the regular English. The paper is logically articulated and its structure is very well detailed.

**A6**. The study adheres to the necessary requirements regarding the ethics of research and experimentation. Following the examination of the document, critical remarks by reviewers and revision comments by authors were dealt with accordingly with the technical problems being fixed.

**A7**. The article follows the right reporting conventions and research ethics concerning data publication.

Hence, **I would advise that the paper can be published** in PLOS ONE journal.
