# Supplementary material for: The impact of new urbanization on “quantity increase and quality improvement” of urban green innovation
Source: PLoS One. 2026 May 19;21(5):e0329052. doi: 10.1371/journal.pone.0329052 (PMC13186382; doi:10.1371/journal.pone.0329052)
Supplement: Attachment — Submitted filename: Response_to_Reviewers_auresp_3.docx [file pone.0329052.s005.docx]

# Response to Reviewers’ Comments

# (Manuscript Number: PONE-D-25-37471R2)

# Dear Reviewers,

We are very grateful for the reviewers’ constructive comments and suggestions on our manuscript entitled “The impact of new urbanization on "quantity increase and quality improvement" of urban green innovation” (ID: PONE-D-25-37471R2). Below, we provide a point-by-point response to each of the reviewers' concerns:

***Response to Reviewer #5 Comments***

**Reviewer #5:**

This manuscript empirically examines the impact of new urbanization on the quantity and quality of green innovation using panel data from Chinese cities and multivariate moderating models. The topic is relevant and potentially publishable; however, several areas require clarification or strengthening before acceptance.

**Comment 1:**

1. While the paper clearly states its research objective, the conceptual links between NEU, GI, and moderating variables should be more rigorously theorized. The manuscript should explicitly justify the selection of proxies for GI (green patent applications and green invention patents), including potential measurement biases and alternatives.

**Response:**

We are deeply grateful for the reviewer's insightful and constructive comments. These suggestions are pivotal for enhancing the theoretical depth and methodological rigor of our study. We have carefully addressed each point in the revised manuscript as detailed below.

We agree that a robust theoretical framework is the backbone of our research. In the revised manuscript (primarily in Theoretical analysis and research hypotheses sections), we have significantly expanded our theoretical exposition to build a rigorous logic chain connecting New Urbanization (NEU), Green Innovation (GI), and the five moderating variables.

First, we have further elaborated on the theoretical connection between the two concepts from the perspectives of New Urbanization (NEU) and Green Innovation (GI). We clarify that NEU transcends mere population agglomeration and spatial expansion; it represents a modern urban development paradigm characterized by economic growth, social coordination, and environmental sustainability. Green Innovation (GI), on the other hand, emphasizes the development and upgrading of green technologies aimed at resource recycling and environmental improvement. It encompasses creative activities across multiple dimensions, including technology, management, and institutions. From a conceptual logic standpoint, NEU constitutes the real-world context and demand engine for GI, while also providing an experimental space and an incentive framework for it, making it a key driving force behind Green Innovation. The paper analyzes how NEU facilitates the theoretical link between NEU and GI through aspects such as resource agglomeration, market demand, resource spatial transfer, and industrial integration. Furthermore, we have supplemented the theoretical logic of the relationship between NEU (New Urbanization) and GI (Green Innovation) by incorporating arguments from both Endogenous Growth Theory and the Constraint-Induced Innovation Mechanism.

The following specific content has been added to the first paragraph of the "Theoretical analysis and research hypotheses" section:

*NEU goes beyond population concentration and spatial expansion, embodying a modern urban development paradigm characterized by economic growth, social harmony, and environmental sustainability. NEU represents a systematic reconfiguration of traditional urbanization, which is typically defined by “population mobility and urban expansion.” GI, on the other hand, emphasizes the development and upgrading of green technologies aimed at resource recycling and environmental improvement; it encompasses creative activities across multiple dimensions, including technology, management, and institutional frameworks. From a conceptual perspective, NEU constitutes the practical context and demand engine for GI, while also providing a testing ground and incentive framework for GI, serving as a key driving force behind it.*

In the third paragraph of the "Theoretical analysis and research hypotheses" section, the following content has been specifically added:

*Furthermore, endogenous growth theory holds that knowledge and technological information are the core drivers of sustained economic growth [25]. NEU brings together resources and factors within a specific geographical area to form a high-density, multi-stakeholder innovation network [26]. Various actors interact frequently within innovation networks; the industry-academia-research collaboration system formed within cities facilitates the rapid dissemination of knowledge and information, accelerating the transmission and recreation of technical knowledge, and leading to knowledge spillover effects and continuous innovation [27]. Constrained by the need for sustainable use of the environment and resources, companies are compelled to seek alternative technologies that meet environmental standards, thereby driving the innovation and development of green technologies.*

Second, in the fourth paragraph of the "Theoretical analysis and research hypotheses" section, the following content has been specifically added:

*Digital inclusive finance (DIF) is a form of finance that primarily relies on digital technology to provide convenient financial products and services to various segments of society[28]. NEU cannot function without strong financial support, DIF can provide convenient, low-cost financial services to various stakeholders in NEU, particularly rural migrant workers, enterprises, and service providers[29]。*

*Through the DIF platform, R&D enterprises can easily secure financing to sup-port GI activities—such as green technology R&D and the procurement of green equipment—thereby driving the production of green outcomes [33].*

Third, in the fifth paragraph of the "Theoretical analysis and research hypotheses" section, the following content has been specifically added:

*Environmental regulation theory posits that government intervention in pollution control promotes green innovation in the long run. Government technical support directly reduces the fixed costs and uncertainty associated with green R&D, increases firms’ willingness to invest in R&D in sectors such as new energy, energy conservation, and environmental protection, and ensures that knowledge spillovers generated during the NEU process are more concentrated in environmentally friendly technologies[40].*

*Informatization is the process of applying modern information technology to optimize resource allocation, improve operational efficiency, and drive economic restructuring[42]. Informatization can build online platforms and networks to break geographical restrictions, and has a positive effect on the equalization and popularization of public services[43]. Migrant workers can access convenient and efficient public services, which will help enhance the level of smart urban management and support the development of NEU.*

*Capital, technology, and talent are vital resources for economic development. Economically advanced regions typically possess greater financial resources for the research, development, and implementation of green technology infrastructure. They can increase investment in renewable energy technology and environmental conservation sectors to promote the improvement of urban infrastructure and the enhancement of public services, and promote the output of GI results [46].*

*Urbanization requires large quantities of energy-intensive products such as steel and cement. As rural migrants move into urban areas, their energy consumption primarily consists of commercial energy sources such as electricity and natural gas, leading to an increase in energy consumption[47]. In general, high-energy-consuming cities tend to face greater pressure on energy consumption and environmental pollution [48], and this compels cities to prioritize GI in the process of NEU.*

Fourth, we first acknowledge that the measurement of green innovation (GI) relies on established classification systems based on predefined green innovation lists. Although these are the most widely accepted standards, they may still be subject to bias or fail to capture all green innovation outcomes. Our choice of green patent applications and green invention patents is primarily based on the fact that these two aspects directly align with the core concept of green innovation (GI). This is because green patent data provides a concrete and tangible measure of innovative activity; the number of applications reflects both the effort and intent behind innovation, thus offering a reasonable way to assess both the process and outcomes of green innovation. This approach is consistent with mainstream literature on environmental and ecological innovation (e.g., Pan, X. et al., 2021), which has confirmed that green patent applications and green invention patents serve as reliable proxy variables for green innovation. Furthermore, robustness checks using alternative measures (see Section 4.2 Robustness test) yielded stable results. In the robustness test, the total number of authorized green patents per 10,000 people (NGIN) was used to measure the “incremental” aspect of green innovation, while the total number of authorized green invention patents per 10,000 people (NGNS) was used to measure the “quality improvement” aspect of green innovation.

The following specific content has been added to the "3.1. Indicator design" section of the paper：

*The total amount of green patent applications not only reflects the situation of innovation activities, but also is closely related to the economic and environmental benefits of GI. Green patent data provides a specific and tangible result measurement standard for innovation activities. The number of applications reflects the innovation efforts and intentions. The number of green patent applications can not only better measure the process of GI, but also directly reflect the research and development results of enterprises, scientific research institutions or individuals in the field of green technology. The number of green patent applica-tions is standardized, quantifiable and internationally universal, which is convenient for statistical analysis and horizontal comparison. This choice is consistent with the mainstream literature on environmental innovation and ecological innovation [51,52].*

**Comment 2:**

2. Please provide variance inflation factors (VIF) or correlation matrices to improve credibility.

**Response:**

To further examine multicollinearity from a statistical perspective, this study calculated the Variance Inflation Factor (VIF) values between the independent variable NEU and the control variables FDI, INT, RFV, HCL, OOW, and SCL. The VIF values for all variables were found to be less than 5, indicating that there is no significant multicollinearity issue between NEU and the control variables. To clearly present the results of the multicollinearity test, we have added Table 5 in the paper to display the VIF data, and included the following content at the end of the "3.3. Data sources and descriptive statistics" section:

*In order to further test the multicollinearity, this paper calculates the VIF ( variance inflation factor ) values of the independent variable NEU and the control variables FDI, INT, RFV, HCL, OOW, SCL. As shown in table 5, the VIF values of all variables are less than 5, and the correlation coefficients between each variable are also less than 0.7. It is considered that there is no multicollinearity problem between NEU and control variables.*

*Table 5 The variance inflation factor and correlation coefficient of each variable*

|  |  | *NEU* | *FDI* | *INT* | *RFV* | *HCL* | *OOW* | *SCL* |
| --- | --- | --- | --- | --- | --- | --- | --- | --- |
| *Variance Inflation Factor* | *VIF* | *2.94* | *1.17* | *1.27* | *1.01* | *1.92* | *1.55* | *1.04* |
|  | *1/VIF* | *0.3398* | *0.8536* | *0.7876* | *0.9881* | *0.5211* | *0.6452* | *0.9572* |
| *correlation coefficient* | *NEU* | *1* |  |  |  |  |  |  |
|  | *FDI* | *0.2341* | *1* |  |  |  |  |  |
|  | *TEL* | *0.5943* | *0.3153* |  |  |  |  |  |
|  | *INT* | *0.4203* | *-0.0321* | *1* |  |  |  |  |
|  | *RFV* | *0.0176* | *-0.0254* | *0.0225* | *1* |  |  |  |
|  | *HCL* | *0.6714* | *0.2274* | *0.3228* | *0.0117* | *1* |  |  |
|  | *OOW* | *0.5489* | *0.2205* | *0.2021* | *-0.0250* | *0.2783* | *1* |  |
|  | *SCL* | *0.0850* | *-0.0203* | *0.0772* | *-0.0814* | *0.1467* | *-0.0531* | *1* |

**Comment 3:**

3. The manuscript reports significant coefficients and adjusted R² values but should provide fuller statistical interpretation. Consider reporting standardized coefficients to enhance interpretability.

**Response:**

(1) We sincerely thank the reviewer for the constructive comment. In the revised manuscript, we have expanded the depth of interpretation for the statistical results. In the "4.1 Benchmark regression" section, we explicitly state the impact coefficient of NEU and its significance level, provide a specific explanation of the economic meaning of this coefficient, and elaborate in detail on its practical implications. Specifically, we have added the following content:

*This means that for every 1 unit increase in NEU, GIN increases by an average of 0.7452 units, GNS increases by an average of 0.7892 units, the adj.R2 in E1 and E3 increases from 0.462 to 0.861, and the adj.R2 in E2 and E4 increases from 0.344 to 0.849. The overall fitting degree of the model is improved, and the control variables such as FDI, INT, RFV, HCL, OOW and SCL in the model explain the 'residual variation', which leads to the improvement of the overall fitting degree of the model. This further verifies that NEU is an important driving force for GIN and GNS, and also shows that the model effectively captures more comprehensive driving factors.*

(2) Regarding the "4.4. Moderating effects" section, we have supplemented our analysis by discussing the coefficients, significance levels, and changes in the adjusted R², along with explaining their practical implications. We also added an interpretation of the adjusted R² value, clarifying how changes in the adjusted R² affect the explanatory power of the model. By incorporating p-values, we explained the practical meaning of statistical significance.

Specifically, we have added the following content:

*The adj. R2 in E23 and E24 are 0.903 and 0.891, respectively. The two models fit well. DIF has a complementary moderating effect, and the moderating mechanism of DIF is strongly supported by the sample data. For every unit increase in the moderating variable DIF, the marginal effect of NEU on GIN increases by 0.0025 units, and the marginal effect of NEU on GNS increases by 0.0026 units.*

*The adj. R2 in E25 and E26 are 0.905 and 0.790, respectively. The two models fit well. DIF has a complementary moderating effect. In strong government support cities, for every unit increase in the moderating variable DIF, the marginal effect of NEU on GIN increases by 0.0020 units. In weak government support cities, it increases by 0.0015 units.*

*The adj. R2 in E29 and E30 are 0.921 and 0.882, respectively. The two models fit well. DIF has a complementary moderating effect. In high-information cities, for every unit increase in the moderating variable DIF, the marginal effect of NEU on GIN increases by 0.0018 units, while in low-information cities, it increases by 0.0015 units.*

The specific content can be found in the marked sections of the manuscript.

(3) To enhance the interpretability of the model results, we have standardized the main explanatory variable NEU as well as the dependent variables GIN and GNS, following your suggestion. In the regression analysis based on the standardized variables, we report the standardized regression coefficients, which directly reflect the number of standard deviations the dependent variable changes for each one-standard-deviation change in the independent variable. This facilitates comparison of the relative impact of different variables on the outcome. The regression coefficients in Tables 6–14 have been updated to reflect the standardized regression results, and the modified parts have been marked in the paper.

**Comment 4:**

4. Some interpretations overgeneralize findings beyond the empirical scope. For example, policy recommendations occasionally extend beyond what panel regressions alone can substantiate.

**Response:**

We fully agree with your opinion. Upon careful reflection, we realized that when drafting the Discussion and Recommendations section, we did indeed overanalyze the "association findings based on panel data," and some of the proposed policy recommendations were not sufficiently supported by the empirical analysis results. Therefore, we have narrowed the scope of our conclusions and, in the "6.3. Limitations and future research" section, explicitly stated that the current study is based on calculations and tests using city-level panel data from 2011 to 2022, rather than representing "deterministic and inevitable results." Moreover, we have re-examined all policy recommendations and rewritten part of their content. The specific modifications are as follows:

*We need to make use of the dual role of NEU in promoting GI, scientifically plan urban functional areas, rationally arrange green industries and high-energy-consuming industries, and build specialized green industrial agglomeration areas to provide space carriers for GI. Combined with the quality improvement effect of NEU on GI, it guides the population to gather in green industries, encourages enterprises to carry out green product innovation, and transforms the population advantage of urbanization into the talent and technology advantage of green innovation. Focusing on the synergistic effect of DIF, government science and technology support, informatization and other factors, this paper constructs a digital service platform of green finance, integrates green technology demand and financing information, develops appropriate green credit products, and provides accurate financial support for enterprises and scientific research institutions. Explore the establishment of cross-sectoral information sharing mechanism, promote the cross-regional optimal allocation of financial resources, and amplify the linkage dividend between new urbanization and green finance. In view of the strengthening effect of government green support on innovation effect, it is suggested to maintain a reasonable proportion of fiscal expenditure on environmental protection, reduce the financing cost of green projects through loan subsidies and guiding funds, attract social capital to participate in GI, and provide policy guarantee for NEU to release greater innovation efficiency.*

In addition, we have removed absolute expressions that required certain mandatory measures to be “must” or “should” implemented, replacing them with more flexible wording to better conform to academic norms. Finally, in the "6.3. Limitations and future research" section, we have added a statement emphasizing the need to adopt more rigorous causal inference methods or quasi-experimental designs to further verify the causal relationship between new urbanization and green innovation.

**Comment 5:**

The manuscript is generally intelligible but contains frequent long and repetitive sentences that reduce readability. I would recommend professional language editing and tightening of phrasing in Results interpretation, Discussion, and Policy implication sections.

**Response:**

You rightly pointed out that “there are frequent long sentences and repetitive expressions, which reduce readability.” We fully accept this observation and sincerely apologize for any inconvenience it may have caused during reading. We have conducted systematic language revisions in the following sections: “Theoretical analysis and research hypotheses”, “4.1 Benchmark regression”, “4.4. Moderating effects”, “6.1. Summary”, and “6.2. Theoretical and policy implications”. We have carefully reviewed all complex, lengthy sentences, breaking them down into shorter, logically progressive ones. Repetitive modifiers and unnecessary conjunctions have been removed, and some semantically redundant sentences have been deleted or merged to improve the conciseness and readability of the text. After completing the content revisions, we commissioned two English-language researchers to perform professional language editing and proofreading on the entire manuscript, in order to eliminate grammatical and lexical errors as much as possible. For specific language modifications, please refer to the marked sections in the text.

**Comment 6:**

Some tables are dense and difficult to interpret; improved formatting or summary visualization could enhance accessibility.

**Response:**

Your comment is very well taken. To improve the accessibility of the results, we have substantially reorganized and streamlined the tables in the robustness test section, following your suggestion to “optimize formatting or add summary visualizations.” First, we merged similar robustness test results to reduce the number of tables. Considering that although the testing methods in Table 6 (Robustness Test I), Table 7 (Robustness Test II), and Table 8 (Robustness Test III) differ, the core results—such as the coefficients of the key explanatory variable, significance levels, and adjusted R²—are highly similar, we combined them into two new tables: Table 6 (Robustness Test I) and Table 7 (Robustness Test II). This consolidation avoids redundant presentation of similar results while retaining all critical statistical information, making the tables more concise. In addition, we removed redundant tables. The information from the original “Threshold Test Results Table” can be conveyed through textual description, so we have deleted it to prevent diverting readers’ attention from the main results, and instead present the threshold test results directly in Table 15. We also adjusted the layout of some tables to ensure clear information hierarchy, enabling readers to quickly locate the key results. For the specific modifications, please see the marked sections in the paper.

**Comment 7:**

Please consider expanding engagement with recent international literature beyond region-specific context to strengthen positioning within global research.

**Response:**

We fully agree that situating the study within the global academic context helps highlight its theoretical value and practical significance.

First, in the "1. Introduction" section, we have supplemented the discussion with recent research literature from the past three years, reviewing representative studies published in international journals on the relationship between new urbanization and green innovation. In both the introduction and the literature review, we have added international research from the past five years and included an analysis of the ongoing debates regarding the impact relationship between new urbanization and green innovation. The references focus primarily on literature from the last five years, reflecting our attention to the cutting edge of international research. At the end of the second paragraph of the "1. Introduction" section, we have added the following content:

*Through the review of the existing literature, it is found that most researchers agree that new urbanization can effectively promote green innovation, and a few researchers put forward the view that new urbanization is a potential obstacle to green innovation. Zhao et al., ( 2025 ) proposed that new urbanization faces resource and environmental constraints in the process of rapid development, which may limit the space and effect of green innovation. Especially in areas with poor resource endowments, the role of new urbanization in promoting green innovation is limited.*

We have conducted a review of the existing research landscape and supplemented the discussion by highlighting the limitations of prior studies. Specifically, we have added the following content to the third paragraph of the "1. Introduction" section:

*The existing related research does not systematically analyze the quantity and the quality of GI as independent explanatory variables, and does not clearly dis-tinguish the difference in the driving mechanism between “quantity increase” and “quality improvement”. The “quantity increase” reflects the activity and popularity of GI, and the “quality improvement” reflects the depth and level of GI. Dividing GI into “quantity increase” and “quality improvement” can more comprehensively evaluate the impact of NEU on GI and avoid the one-sidedness of policy effect evaluation, so as to provide a basis for policy formulation more accurately and more pertinently, and also help guide the development direction of GI practice. In the existing research, there is also a lack of systematic analysis based on multi-theoretical perspectives such as urban development theory, GI theory, digital finance theory and agglomeration effect. Most of the moderating factors such as government support and digital inclusive finance are assumed to be linear and lack nonlinear tests such as threshold effects. This limits our comprehensive understanding of GI effect of NEU and weakens the accuracy of policy formulation.*

Finally, we have revised the conclusion to emphasize that this study not only provides policy reference for the construction of new urbanization but also offers new evidence for verifying and extending international research on the interaction mechanisms among urbanization, digital finance, and green innovation. In the "6.2. Theoretical and policy implications" section, we have modified the discussion of the theoretical implications. The specific content is as follows:

*Our research focuses on the impact of NEU on urban GI, challenging the tradi-tional view that urbanization focuses on economic growth and resource utiliza-tion. NEU emphasizes green development and ecological benefits, and its role in promoting GI is more obvious, which is in line with the new urban development theory. It provides a new theoretical perspective for how urbanization promotes GI and enriches the existing theory of the relationship between urbanization and innovation. This paper discusses the ' incremental effect ' and ' quality improvement effect ' of NEU on GI, expands the theoretical framework of GI research from one-dimensional to two-dimensional, and enriches the research results of NEU. DIF amplifies the effect of NEU on urban GI, revealing its nonlinear characteristics and multivariate synergistic regulation mechanism (digital inclusive finance, government technology support, informatization, re-gional economy, energy consumption). This is not only the deepening of Porter hypothesis and endogenous growth theory in the context of green transformation, but also helps to reveal the internal logic and complex mechanism of NEU affecting urban GI more comprehensively and deeply. It also provides a new theoretical explanation dimension for understanding how the institutional, financial and technological environment jointly shape the impact path of urban-ization on innovation. This study not only provides policy reference for the construction of NEU, but also provides important empirical evidence and new analytical perspective for the development of urbanization-green innovation theory with the characteristics of ' transition economies ', and provides new ev-idence for verifying and expanding international research on the interaction mechanism of urbanization, digital finance and green innovation. Compared with the relevant international research, Existing research mainly analyzes that NEU can promote urban GI from the perspective of enterprises [78], policy [79,80] and green development [81]. However, the urbanization models in different regions are different, and the impact of different urbanization models on regional green technology R&D are also different [8]. Moreover, the impact of NEU on GI also needs to be played through mediating variables. For example, NEU promotes GI by alleviating corporate financial constraints [9]. In addition, existing international research often considers NEU as an independent driving factor into the innovation system for research. This study not only analyzes the “quantity increase” and “quality improvement” effects of NEU and urban GI, but also incorporates NEU with DIF, GTS, INT, AGDP, and ENC into the same analytical framework, revealing the complex mechanism, which provides a new perspective for understanding the relationship between NEU and GI in different contexts.*

**Comment 8:**

Highlight how the contribution advances theory rather than primarily confirming existing findings.

**Response:**

We have restructured the "Empirical Analysis" conclusions and the "Research Contributions" section to clearly articulate how this work advances, rather than merely confirms, existing theories. The specific modifications mainly focus on the following aspects:

(1) The discussion of the benchmark regression results now highlights how the findings complement and differ from existing theories. It is argued that this conclusion, for the first time, systematically deconstructs the quantitative expansion and qualitative improvement of green innovation within a unified framework. This expands the theoretical boundaries of the urbanization–innovation relationship, enabling a more precise explanation of the real-world demand in green transformation to “pursue both quantity and quality.”

(2) In terms of theoretical contributions, we have analyzed how the study extends and deepens existing theories. It points out that previous related research has not treated the two aspects as independent and parallel dependent variables for systematic analysis, nor has it clearly distinguished the differences in driving mechanisms between “incremental” and “qualitative improvement” effects. For example, agglomeration economy theory and growth pole theory have mostly focused on the linear increase of innovation “quantity,” while regional development theories generally discuss “innovation” in broad terms. The “incremental effect” and “qualitative improvement effect” of new urbanization on green innovation expand the theoretical framework of green innovation research from a unidimensional to a dual-dimensional perspective, thereby enriching the results of research on new urbanization.

(3) Most existing studies on urbanization and green innovation remain at the stage of verifying linear relationships or examining single moderating effects. There has been relatively little exploration of multidimensional moderating effects or the nonlinear mechanisms through which new urbanization influences green innovation. This study not only confirms the positive effect of new urbanization on green innovation but also reveals its nonlinear characteristics and the synergistic moderating mechanisms involving multiple variables—namely, digital inclusive finance, government support for science and technology, informatization, regional economy, and energy consumption. This contributes to a more comprehensive and in-depth understanding of the internal logic and complex mechanisms underlying the impact of new urbanization on green innovation. It also offers a new theoretical explanatory dimension for understanding how institutional, financial, and technological environments jointly shape the pathways through which urbanization affects innovation.

(4) We have added a discussion on how this study contributes to research on emerging economies. Based on China’s rapid urbanization and economic transition, the study provides important empirical evidence and a novel analytical perspective for developing a urbanization–green innovation theory that incorporates the characteristics of “transitional economies.”

In the "1. Introduction" section, the research contributions in the third paragraph have been rewritten as follows:

*(1) Agglomeration economic theory and growth pole theory mostly focus on the linear growth of quantity, and regional development theory also mainly discusses innovation in a broad sense. This paper focuses on the relationship between NEU and GI, empirically analyzes the impact of NEU on GI, and specifically discusses the "quantity increase" and "quality improvement" effect of NEU on GI, which expands the theoretical framework of GI research from one-dimensional to two-dimensional, enriches the research results of NEU.*

*（2）The existing research on NEU and GI is mainly reflected in the verification of linear relationship or single moderating effect. There are few studies on the following mechanisms of the multi-dimensional moderating effect and the non-linear relationship between NEU and GI. This study not only verifies the positive effect of NEU on GI, but also reveals its nonlinear characteristics and multivariate co-regulation mechanism ( digital inclusive finance, government technology support, informatization, regional economy, energy consumption ). This is not only a deepening of Porter 's hypothesis and endogenous growth theory in the context of green transformation, but also provides a new theoretical explanation dimension for understanding how institutional, financial and technological environments jointly shape the impact of urbanization on innovation, and expands the content of urban development theory and GI theory.*

*（3）Different from the existing literature, which usually only examines the single moderating variable or the full sample moderating effect, this paper designs a multi-dimensional grouped moderating effect analysis framework, which enables the simultaneous identification of multiple groups and multiple sources of heterogeneity after controlling for cities and years effects. The multivariate moderating effect helps to reveal the internal logic and complex mechanism of NEU affecting GI more comprehensively and deeply, and provides new ideas for future research.*

*(4) Much of the international research is based on the context of developed countries and focuses on urbanization and innovation in the post-industrial era. This paper, however, combines an analysis of China’s NEU strategy—proposed in the new era—with the specific historical context of “economic transformation and development.”From the standpoint of government green support, this paper verifies that the relationship between NEU and GI is not a linear trend, which further enhances comprehension of the complex influence relationship between NEU and GI. This provides important empirical evidence and new analytical perspectives for the development of NEU and GI theories.*

In section 4.1 Benchmark regression, the following content has been added:

*This shows that China is shifting from the traditional ' scale-driven ' growth to the ' innovation-driven ' development in the new era. Urbanization is no longer a simple population migration. NEU is actively exerting the effect of factor agglomeration and high-quality innovation, and guiding the industry to the direction of green, low-carbon and modernization. This also verifies the viewpoint of endogenous growth theory and innovation theory, and the spatial agglomeration of economic activities effectively promotes innovation activities[54]. The industrial upgrading and environmental regulation brought by NEU have forced enterprises to actively seek fundamental changes and GI to gain a competitive advantage. As a key link, the “quality improvement” of GI can better reflect the deep value of NEU than the “quantity increase”.*

*This conclusion systematically deconstructs the quantity increase and quality improvement of urban GI under the same framework for the first time, which expands the theoretical boundary of urbanization-innovation relationship and makes it more accurate to explain the practical needs of ' quantity and quality ' in green transformation. H1 is verified.*

In the "6.2. Theoretical and policy implications" section of the paper, we have rewritten the theoretical implications, as detailed below:

*Our research focuses on the impact of NEU on urban GI, challenging the tradi-tional view that urbanization focuses on economic growth and resource utiliza-tion. NEU emphasizes green development and ecological benefits, and its role in promoting GI is more obvious, which is in line with the new urban development theory. It provides a new theoretical perspective for how urbanization promotes GI and enriches the existing theory of the relationship between urbanization and innovation. This paper discusses the ' incremental effect ' and ' quality improvement effect ' of NEU on GI, expands the theoretical framework of GI research from one-dimensional to two-dimensional, and enriches the research results of NEU. DIF amplifies the effect of NEU on urban GI, revealing its nonlinear characteristics and multivariate synergistic regulation mechanism (digital inclusive finance, government technology support, informatization, re-gional economy, energy consumption). This is not only the deepening of Porter hypothesis and endogenous growth theory in the context of green transformation, but also helps to reveal the internal logic and complex mechanism of NEU affecting urban GI more comprehensively and deeply. It also provides a new theoretical explanation dimension for understanding how the institutional, financial and technological environment jointly shape the impact path of urban-ization on innovation. This study not only provides policy reference for the construction of NEU, but also provides important empirical evidence and new analytical perspective for the development of urbanization-green innovation theory with the characteristics of ' transition economies ', and provides new ev-idence for verifying and expanding international research on the interaction mechanism of urbanization, digital finance and green innovation. Compared with the relevant international research, Existing research mainly analyzes that NEU can promote urban GI from the perspective of enterprises [78], policy [79,80] and green development [81]. However, the urbanization models in different regions are different, and the impact of different urbanization models on regional green technology R&D are also different [8]. Moreover, the impact of NEU on GI also needs to be played through mediating variables. For example, NEU promotes GI by alleviating corporate financial constraints [9]. In addition, existing international research often considers NEU as an independent driving factor into the innovation system for research. This study not only analyzes the “quantity increase” and “quality improvement” effects of NEU and urban GI, but also incorporates NEU with DIF, GTS, INT, AGDP, and ENC into the same analytical framework, revealing the complex mechanism, which provides a new perspective for understanding the relationship between NEU and GI in different contexts.*

**Comment 9:**

How sensitive are the results to alternative measures of green innovation or urbanization indicators and to different fixed-effects structures or clustering strategies?

**Response:**

We fully agree that it is crucial for the empirical analysis results in the paper to be insensitive to model specification and variable measurement. As the authors, we share this concern and repeatedly verified during the data calculation process whether the analysis results were sensitive to the model and variables. In this study, we employed five different robustness check methods—Removing special city samples, Removing special year samples, Adding dummy variables, Replacing independent variable, and Replacing dependent variable—as well as two endogeneity test methods: the lagged variable approach and the instrumental variable approach. The test results indicate that the empirical analysis findings are robust. We have provided further explanations for the rationale behind selecting these robustness check methods.（1）Removing special city samples. The special city samples selected in this study were not determined subjectively by the authors. Instead, the four municipalities directly under the central government in China—Beijing, Chongqing, Tianjin, and Shanghai—were treated as special city samples for analysis. This decision was made considering that these four cities are leaders in China’s economic development and major hubs of innovation agglomeration, with annual numbers of innovation achievements consistently ranking among the highest nationwide. Consequently, including these four cities may have a significant influence on the innovation effects of new urbanization. To test the robustness of the benchmark regression results, we excluded these four cities from the sample and performed a robustness check; the results remained robust.（2）Removing special year samples. The selection of years was not based on subjective judgment or random sampling. China formally introduced its new urbanization strategy in 2015. Taking into account the time lag in the effects of policy implementation, the government further rolled out the strategy nationwide in 2016. To avoid potential interference from these two years’ samples on the regression results, this study excluded the data from 2015 and 2016 for the robustness check. The results remained robust.（3）Adding dummy variables. We added the binary (0/1) dummy variable the new-energy model cities policy (NEM)to the model, which effectively controls for the exogenous shock of “whether this policy was implemented” separately. After controlling for this policy, the coefficient of NEU remained largely unchanged in direction, magnitude, and statistical significance, thereby enhancing the robustness of our conclusions. The reason for selecting this policy is that the new-energy model cities policydirectly targets green development and the new energy industry, making it highly relevant to green innovation. It is also one of the most representative city-level environmental regulation policies since 2014. Incorporating this policy into the regression model helps absorb region-specific characteristics associated with such policies to some extent, and it controls for potential exogenous impacts arising from resource tilting, institutional support, and other factors linked to the policy. This allows us to test whether the original results are sensitive to these characteristics. After controlling for the effect of the new-energy model cities policy, the positive effect of NEU on GI remains robust. This indicates that the mechanism through which NEU promotes GI does not depend on the implementation of this particular policy, but instead possesses broader explanatory power. (4) Replacing independent variable. In this study, we use the entropy method to calculate a composite score for NEU, which serves as the independent variable in the regression analysis. However, every measurement method has its limitations. It is well known that the entropy method is relatively sensitive to outliers, whereas factor analysis handles multicollinearity among indicators more effectively. To test whether the conclusions are influenced by a specific weighting method, we compared the results obtained from the two approaches. If the impact results remain highly consistent in both statistical significance and economic meaning under the two measurement methods, it can effectively demonstrate that the observed effect is rooted in the data itself, rather than being determined by a particular algorithm.（5）Replacing dependent variable. To address the potential impact of the gap between the number of patent applications and granted patents on the measurement of green innovation, we employed two alternative indicators—namely, the number of green patent applications and the total number of granted green patents—to measure green innovation and examine the effect of NEU on GI. Under all alternative measurements, the estimated coefficient of the NEU variable remained positive and statistically significant. This indicates that our main findings are not driven by a specific proxy variable for green innovation.

Based on the explanations provided above regarding the relevant robustness checks, we have further revised and refined the content of the "4.2 Robustness test" section. For specific details, please refer to the marked portions in the manuscript.

**Comment 10:**

What evidence supports the validity and strength of instruments used? Please Clarify.

**Response:**

Thank you for raising the important issue regarding the validity of the instrumental variable. We fully understand that addressing endogeneity is crucial to the credibility of our analysis. In the revised manuscript, we have strengthened the discussion and reporting of this issue (please see Section 4.3 Endogenous analysis for details). Our response focuses on the two key conditions for instrumental variable validity: relevance and exogeneity.

This study employs two instrumental variables for the endogenous analysis: The independent variable lagged one period (LAG_NEU) and Night lighting value (NLV). First, regarding the instrumental variable The independent variable lagged one period (LAG_NEU), to enhance transparency, we present both the first-stage and second-stage regression results in the manuscript. In the first-stage regression, the coefficient of the instrumental variable passes the significance test at the 0.01 level, satisfying the relevance requirement. As for the exogeneity requirement of the instrumental variable, we have supplemented the theoretical justification. Specifically, we have expanded the section “i) The independent variable lagged one period (LAG_NEU) is used as an instrumental variable” to provide a more detailed theoretical basis. We argue that LAG_NEU is a predetermined variable. Due to the irreversibility of causal timing, the level of urbanization in period t−1cannot be influenced by unforeseeable shocks occurring randomly in period t. Therefore, LAG_NEU satisfies the core assumption of exogeneity for an instrumental variable—that is, it is uncorrelated with the error term in the current period. Additionally, we include city fixed effects and year fixed effects in the model to control for the possibility that city-specific characteristics may be correlated with both LAG_NEU and GI, which further strengthens the theoretical foundation for treating LAG_NEU as a valid instrumental variable.

We further provide statistical support for the exogeneity of our instrumental variable through statistical tests. The LM statistic is 639.126 (p < 0.01), confirming that the instrumental variable satisfies the relevance requirement with respect to the endogenous variable. The Cragg-Donald Wald F-statistic is 820.614, which exceeds the threshold of 15, ruling out the possibility of weak instruments. Therefore, we have added the following discussion to the section “i) The independent variable lagged one period (LAG_NEU) is used as an instrumental variable”:

*Since time is irreversible, the predetermined variable is not affected by the cur-rent period [63]. LAG_NEU is a predetermined variable, NEU in t-1 period cannot be affected by t period. There is no reverse causality between GI and LAG_NEU. LAG_NEU is not related to the current error term. LAG_NEU satisfies the core hypothesis of instrumental variable exogeneity. At the same time, cities fixed effects and years fixed effects are introduced into the model to control the problem that urban characteristics may be related to both LAG_NEU and GI. While LAG_NEU may be correlated with past realizations of the endogenous variable, it satisfies the exclusion restriction as it influences the current outcome solely through its impact on the contemporaneous level of NEU.*

*The statistical value of Anderson canon.corr.LM statistic is 639.126, p<0.01, underidentification test passes, the statistical value of Cragg-Donald Wald F =820.614>15, and weak instrumental variable test passes.*

Secondly, regarding the instrumental variable Night lighting value (NLV), we have provided a more comprehensive theoretical justification and result presentation in the revised manuscript. From a theoretical perspective, we explain why NLV can serve as a valid instrument. Higher night light values often indicate greater population agglomeration in a given area, which in turn tends to reflect a higher level of urbanization. The exogeneity of NLV is rooted in its unique nature as a satellite-based remote sensing measure of human activity. NLV primarily captures light emissions from infrastructure, industrial production, and commercial activities, which are directly observed via satellite imagery and are generally unaffected by local government policy interventions or manipulation of economic indicators. Moreover, we point out that, apart from its relationship with NEU, NLV has almost no other direct or significant channels through which it could influence GI, making it an exogenous variable. Existing studies have frequently used NLV as a proxy or instrumental variable for NEU in endogeneity analysis (e.g., Sharma, N., Shastri, S., & Shastri, S., 2023; Pambuku, A. et al., 2024). From a statistical standpoint, we conducted and reported two key tests. The coefficient of NLV passed the significance test at the 0.01 level. The Anderson canonical correlation LM statistic is 36.334 (p < 0.01), providing strong evidence to reject the null hypothesis of “underidentification.” This confirms that our instrumental variable, night light value (NLV), is statistically correlated with the endogenous variable new urbanization (NEU). The Cragg-Donald Wald F-statistic is 36.551, which is greater than 15, providing strong evidence that our instrument is not weak, and indicating that the Two-Stage Least Squares (2SLS) estimates are unlikely to suffer from serious weak-instrument bias. We have provided statistical evidence to demonstrate the strength of the instrumental variable, as well as a clear theoretical basis for its exogeneity. The combination of statistical test results and our theoretical arguments gives us confidence that the instrumental variable approach adopted in this paper is valid, and that the resulting estimates are reliable.

Therefore, building upon the existing analysis, we have supplemented the section “ii) Night lighting value (NLV) as an instrumental variable” with additional theoretical justification and supporting literature. The specific content is as follows:

*NLV has natural properties and mainly captures light emission from infrastructure, industrial production and commercial activities. It is generally directly observed by satellite remote sensing. NLV reflects the objective geographical distribution of energy consumption and human activities, and is not affected by local government policy intervention or economic activities [65]. Moreover, the causal relationship between the impact of NLV on the environ-ment and GI activities such as technological progress and R&D investment is extremely weak and indirect, and does not have a direct impact on GI, which is an exogenous variable. Most of the existing researchers use NLV as a proxy variable or instrumental variable of NEU for endogenous analysis [66,67].*

**Comment 11:**

The manuscript relies heavily on reduced-form econometric validation. Could the authors clarify how their findings advance causal inference rather than association-based policy recommendation?

**Response:**

You correctly pointed out that relying solely on reduced-form analysis is insufficient, and we fully recognize the importance of establishing causality for deriving meaningful policy recommendations. Accordingly, we have carefully revised the manuscript with regard to language expression, the argumentation of analytical results, and the discussion of conclusions, in order to strengthen our causal claims. The main modifications are summarized as follows:

First, regarding language. We have systematically reviewed and revised the existing language throughout the manuscript to the greatest extent possible, reshaping the conclusions using causal language. In the Abstract, Introduction, Empirical Analysis, Conclusion, and Discussion sections, we have modified the wording, rewriting certain sentences and removing descriptions of mere correlations, explicitly defining them as causal effects. Examples are as follows:

“This verifies that NEU not only has a positive effect on the “quantity increase” of GI but also significantly contributes to the “quality improvement” of GI” is rewritten as “The evidence suggests that NEU serves as a catalyst for green innovation, positively impacting both “quantity increase” and “quality improvement””

“Examine the influence relationship between NEU and GI using endogeneity analysis” is rewritten as “Therefore, by employing an instrumental variable approach to address endogeneity, we identify a causal effect of NEU, which leads to a significant increase in the quantity and quality of green innovation.”

“It is found that NEU not only has a positive contribution to the quantity increase of GI but also plays a positive role in the quality improvement of GI” is rewritten as “Evidence from the analysis suggests that NEU acts as a catalyst for GI, positively fostering the quantity increase and the quality improvement. ”

“NEU has a positive effect on GI” is rewritten as “The empirical results show that NEU promotes GI”；

“There is a positive correlation between NEU and GI” is rewritten as “Given the causal evidence that NEU drives GI”.

Second, we employed a two-way fixed effects model to examine the effect of NEU on GI, and applied the instrumental variable (IV) approach to conduct endogeneity analysis on the benchmark regression results. For the endogeneity analysis, we used The independent variable lagged one period (LAG_NEU) and Night lighting value (NLV) as instrumental variables. We expanded the theoretical justifications for both relevance and exogeneity, strengthening the foundation for interpreting our estimation results causally, and confirmed the plausibility of the endogeneity assumptions through statistical tests.

**Comment 12:**

What is the manuscript’s core methodological contribution beyond application of established panel techniques to a new dataset?

**Response:**

We thank the reviewer for raising this important point. Beyond applying established panel techniques to a new dataset, the manuscript’s core methodological contribution lies in the following aspects: (1)We develop a multi-dimensional grouped moderation framework: after controlling for unobserved individual and time heterogeneity via two-way fixed effects, we systematically examine the moderating role of DIF on NEU and GI across four theoretically motivated grouping variables—GTS, INT, AGDP, and ENC. (2)Unlike much of the existing literature that focuses on either full-sample moderation or a single moderator, this study implements simultaneous identification of multiple sources of heterogeneity within a panel setting, accompanied by inter-group coefficient difference tests to assess whether moderation effects vary significantly across groups. (3)This combined strategy provides a replicable econometric approach for disentangling how different factors differentially shape causal relationships, while maintaining robustness through fixed effects control.

In the revised manuscript, we have added a dedicated subsection in the third paragraph of “1. Introduction” section to highlight these contributions, and further discussed their value for multi-dimensional heterogeneity analysis in the Conclusion.

The details are as follows:

*Different from the existing literature, which usually only examines the single moderating variable or the full sample moderating effect, this paper designs a multi-dimensional grouped moderating effect analysis framework, which enables the simultaneous identification of multiple groups and multiple sources of heterogeneity after controlling for cities and years effects. The multivariate moderating effect helps to reveal the internal logic and complex mechanism of NEU affecting GI more comprehensively and deeply, and provides new ideas for future research.*

**Once again, thank you for your professional guidance. We will continue to improve the quality of the manuscript.**

***Response to Reviewer #6 Comments***

**Reviewer #6:**

I am writing to provide my review of the manuscript entitled “The Impact of New Urbanization on the Quantity and Quality of Green Innovation: Evidence from Multivariate Moderating Effect Models.” This paper examines whether new urbanization in China promotes both the quantity and the quality of green innovation using panel data from 284 cities during 2011–2022. The findings indicate that new urbanization significantly enhances green innovation, with digital inclusive finance and government-related factors further strengthening this positive effect.

**Response:**

We sincerely appreciate your careful and thorough review. We fully agree with your suggestions and have made specific revisions and improvements in the revised manuscript. Below is our point-by-point response.

**Comment 1:**

1. Consider adopting a more concise and conceptually integrated title.

**Response:**

We agree with your viewpoint and have revised the paper’s title to “**The impact of new urbanization on ‘quantity increase and quality improvement’ of urban green innovation**”. The revision is primarily based on the following considerations: (1) Removing “based on a multivariable moderation effect model”makes the title more concise. (2) Highlighting the “dual effect” of new urbanization on green innovation clarifies the theme, emphasizing that new urbanization simultaneously influences both the scale and the quality of green innovation. (3) Both “quantity increase”and “quality improvement”are commonly used terms, which facilitate comprehension, interdisciplinary communication, and retrieval.

**Comment 2:**

2. Abstract

•Add information on model types, sample size, and the direction and magnitude of key coefficients.

•Reduce repetition and tighten the wording.

**Response:**

In accordance with the comments, we have made the following revisions:

(1) Added information on model type, sample size, and key coefficients:

The Abstract now specifies that we employed Two-way fixed effects model and multivariate moderation effects models based on a sample of N =284 observations. We explicitly report the direction and significance level of the core coefficients related to the effects of New Urbanization on Quantity Increase and Quality Improvement of Green Innovation (GI).

(2) Reduced repetition and tightened wording:

We removed the redundant sentence: “Digital inclusive finance will further unleash the development dividends brought by NEU and fully leverage the positive effects of GI.” The descriptions of moderating effects among Digital Inclusive Finance, Government Support, Informatization, Regional Economic Development, and Energy Consumption have been consolidated into a single concise statement, avoiding unnecessary repetition while preserving all essential information.

The original phrasing — “The stronger the government's green support, the more conducive it is to new urbanization to further exert quantity increase and quality improvement effects on GI”— has been revised to: “Threshold effect analysis indicates that government’s green support exerts a threshold effect on the influence of NEU over GI.” This formulation shortens the text and highlights the methodological finding.

These adjustments improve clarity, conciseness, and completeness of the Abstract. The revised version is provided below:

*New urbanization (NEU) is a major national strategy that China has proposed and im-plemented. Whether NEU can promote quantity increase and quality improvement of green innovation (GI) is a common concern of the government and scholars. Based on the panel data of 284 cities from 2011 to 2022, this study constructs two-way fixed effects models and multivariate moderation effects models, and empirically analyzes the effect of NEU on the “quantity increase” and “quality improvement” of GI. The study finds that the coefficients for NEU’s impact on “quantity increase” and “quality improvement” of GI are statistically significant at p<0.01, with positive directions indicating enhancement effects. The moderating effect indicates the interaction of digital inclusive finance and government support, informatization, regional economic development, energy consumption all amplify the development dividends of NEU and exert strong "quantity increase" and "quality improvement" effects of GI. Threshold effect analysis indicates that government’s green support exerts a threshold effect on the influence of NEU over GI. The conclusions of this paper have important policy implications for further promoting the construction of new urbanization and GI.*

**Comment 3:**

3.Introduction

•Include a dedicated research-gap paragraph explicitly stating what previous studies have overlooked and why distinguishing between innovation quantity and quality is important.

•Strengthen the theoretical framing (e.g., innovation economics, environmental regulation theory, agglomeration theory, and digital finance theory).

•Reduce policy-oriented narrative and increase analytical synthesis of prior literature.

**Response:**

Thank you for your constructive comments. Following your suggestions, we have made the following revisions:

(1) We have added a new paragraph on research gaps after the second paragraph of the Introduction. This paragraph explicitly states that existing studies mainly examine the impact of new urbanization on the overall level of innovation, while few have simultaneously investigated the two dimensions of green innovation—quantity and quality. In particular, there is a lack of systematic analysis grounded in multiple theoretical perspectives, such as urban development theory, green innovation theory, digital finance theory, and agglomeration effects. Moreover, most studies treat moderating factors such as government support and digital inclusive finance under linear assumptions, without conducting nonlinear tests such as threshold effect analysis. These limitations hinder a comprehensive understanding of the green innovation effects of new urbanization and weaken the precision of policymaking.

The specific content added is as follows:

*The existing related research does not systematically analyze the quantity and the quality of GI as independent explanatory variables, and does not clearly dis-tinguish the difference in the driving mechanism between “quantity increase” and “quality improvement”. The “quantity increase” reflects the activity and popularity of GI, and the “quality improvement” reflects the depth and level of GI. Dividing GI into “quantity increase” and “quality improvement” can more comprehensively evaluate the impact of NEU on GI and avoid the one-sidedness of policy effect evaluation, so as to provide a basis for policy formulation more accurately and more pertinently, and also help guide the development direction of GI practice. In the existing research, there is also a lack of systematic analysis based on multi-theoretical perspectives such as urban development theory, GI theory, digital finance theory and agglomeration effect. Most of the moderating factors such as government support and digital inclusive finance are assumed to be linear and lack nonlinear tests such as threshold effects. This limits our comprehensive understanding of GI effect of NEU and weakens the accuracy of policy formulation.*

(2) We have further elaborated on the importance of distinguishing between the quantity and quality of innovation. Considering the differences in connotation between "quantity increase" and "quality improvement," their respective significance for green innovation also varies. We believe that dividing green innovation into these two dimensions not only allows for a more precise identification of the impact effects of new urbanization but also facilitates the provision of more accurate evidence for policy formulation.

We have added the following content to the third paragraph of the Introduction:

*The “quantity increase” reflects the activity and popularity of GI, and the “quality improvement” reflects the depth and level of GI. Dividing GI into “quantity increase” and “quality improvement” can more comprehensively evaluate the impact of NEU on GI and avoid the one-sidedness of policy effect evaluation, so as to provide a basis for policy formulation more accurately and more pertinently, and also help guide the development direction of GI practice.*

(3) In the fourth paragraph of the Introduction, we have supplemented relevant discussions on urban development theory, green innovation theory, digital finance theory, and endogenous growth theory, and explained how these theories collectively support the analytical framework and research hypotheses of this paper. For example: “expanding the theoretical framework of green innovation research from a unidimensional to a dual-dimensional perspective”; “this study provides a new theoretical explanatory dimension and enriches the content of both urban development theory and green innovation theory”; “endogenous growth theory posits that knowledge and information are core elements driving sustained economic growth. NEU concentrates resources and factors within a specific spatial scope, forming a high-density, multi-agent innovation network.”; “This offers important empirical evidence and a novel analytical perspective for developing an urbanization–green innovation theory with characteristics of a ‘transitional economy.’

In the section “2. Theoretical analysis and research hypotheses,” we have added content explaining how the relevant theories support the research framework of this study, such as: “Environmental regulation theory suggests that government intervention promotes the development of green innovation in enterprises and regions in the long run” and “Digital finance theory argues that financing constraints limit resource allocation and firms’ innovation activities.”

(4) We have reduced the narrative related to policy orientation in the original text, moving some policy-related analyses to the Conclusion section. Considering that many language revisions are scattered across different parts of the manuscript, we have not listed the specific revised content here; all changes have been marked directly in the manuscript.

**Comment 4:**

4.Research Hypotheses

•Explicitly link the hypotheses to established theoretical models.

•Provide a conceptual framework figure illustrating the causal pathways.

•Clarify whether the moderating effects are complementary, substitutive, or threshold-based.

**Response:**

Thank you for your detailed suggestions regarding the research hypotheses section. We have revised the “2. Theoretical analysis and research hypotheses” part based on the following aspects:

(1) Before each research hypothesis, we have supplemented the theoretical basis on which it relies. For example, in the theoretical analysis of hypothesis 1, we introduced the factor agglomeration theory, proposing that the spatial agglomeration of factors forms a regional economic layout with clear division of labor and complementary advantages, where frequent interactions among enterprises can lead to more effective innovation.

We have also incorporated the endogenous growth theory, and added the following content in the theoretical analysis of hypothesis 1：

*Furthermore, endogenous growth theory holds that knowledge and technological information are the core drivers of sustained economic growth [25]. NEU brings together resources and factors within a specific geographical area to form a high-density, multi-stakeholder innovation network [26]. Various actors interact frequently within innovation networks; the industry-academia-research collaboration system formed within cities facilitates the rapid dissemination of knowledge and information, accelerating the transmission and recreation of technical knowledge, and leading to knowledge spillover effects and continuous innovation [27]. Constrained by the need for sustainable use of the environment and resources, companies are compelled to seek alternative technologies that meet environmental standards, thereby driving the innovation and development of green technologies.*

In the theoretical analysis section of hypothesis 3, we have introduced environmental regulation theory to re-examine and analyze the relationship among government intervention, knowledge spillover from new urbanization, and firms’ green innovation behavior, and have added the following content to the text:

*Environmental regulation theory posits that government intervention in pollution control promotes green innovation in the long run. Government technical support directly reduces the fixed costs and uncertainty associated with green R&D, increases firms’ willingness to invest in R&D in sectors such as new energy, energy conservation, and environmental protection, and ensures that knowledge spillovers generated during the NEU process are more concentrated in environmentally friendly technologies[40].*

(2) Conceptual Framework Diagram

At the end of the “2. Theoretical analysis and research hypotheses” section, we have added an influence relationship diagram (Figure 1: Influence Relationship Diagram) showing the relationships among the main variables—new urbanization (NEU), green innovation (GI), digital inclusive finance (DIF), government technology support (GTS), informatization (INT), regional economic development (AGDP), and energy consumption (ENC). This figure visually presents the main effect pathways through which new urbanization affects the quantity and quality of green innovation, and indicates the positions and influence paths of each moderating variable. The moderating effects examined in this study include both single-variable moderating effects (e.g., the moderating effect of DIF on the relationship between NEU and GI) and multivariable moderating effects (i.e., the interaction moderating effects of DIF with GTS, INT, AGDP, and ENC, respectively), represented as DIF×GTS, DIF×INT, DIF×AGDP, and DIF×ENC.

The specific content added is as follows:

*Based on the above analysis, this paper identifies the primary pathways through which NEU influences GI, as well as the effects of moderating varia-bles—including DIF, government support for technology (GTS), informatization (INT), regional economic development (AGDP), and energy consumption (ENC)—on NEU and GI. The relationships among these variables are illustrated in Figure 1 (where DIF×GTS, DIF×INT, DIF×AGDP, and DIF×ENC denote interaction-mediation effects).*

*Figure 1. The influence path diagram of variables*

(3) Types of Moderating Effects

Based on existing studies, common types of moderating effects include complementary, weakening, reversal (direction change), and threshold effects. When the coefficients of the main effect and the interaction term have the same sign, it can be interpreted that the moderating variable and the independent variable exhibit a complementary effect.

In this study, the interaction term between digital inclusive finance (DIF) and new urbanization (NEU) (NEU×DIF) has a coefficient that is significant at the 0.01 level. Subsequently, subgroup tests were conducted using four variables: government technology support (GTS), informatization (INT), per capita GDP (AGDP), and energy consumption (ENC). The coefficients of the NEU×DIF interaction term remain positive and significant at the 0.01 level across all subgroups, indicating that the presence of the moderating variables strengthens the positive effect of new urbanization on green innovation. Therefore, the moderating effect observed in this study is a complementary effect.

In the “2. Theoretical analysis and research hypotheses” section, we have clarified the type of moderating effect in this study, and have made corresponding additions in the following subsections:i) Moderating effect of DIF,ii) Moderating effect of DIF and GTS,iii) Moderating effects of DIF and INT,iv) Moderating effects of DIF and AGDP, v) Moderating effects of DIF and ENC.

The specific supplementary content has been marked in the manuscript.

**Comment 5:**

5.Research Design

•Report VIF statistics and correlation diagnostics.

•Provide clearer transparency regarding data preprocessing procedures.

**Response:**

According to your comments, we have made the following supplements in the revised manuscript:

(1) Variance Inflation Factor (VIF) and correlation diagnostics. At the end of the “3.3. Data sources and descriptive statistics” section in the revised version, we have added a report of the VIF values for all independent and control variables. The results show that there is no multicollinearity issue among the variables, as all VIF values are well below the commonly used threshold of 10.

(2) Correlation diagnostics. We analyzed the correlation coefficients between the independent variables to more clearly present the pairwise correlations among the main predictors. All correlation coefficients are below 0.7, which also supports the conclusion that there is no multicollinearity problem among the variables.

A new paragraph has been added to the “3.3. Data sources and descriptive statistics” section, as follows:

*In order to further test the multicollinearity, this paper calculates the VIF ( var-iance inflation factor ) values of the independent variable NEU and the control variables FDI, INT, RFV, HCL, OOW, SCL. As shown in table 5, the VIF values of all variables are less than 5, and the correlation coefficients between each variable are also less than 0.7. It is considered that there is no multicollinearity problem between NEU and control variables.*

*Table 5 The variance inflation factor and correlation coefficient of each variable*

|  |  | *NEU* | *FDI* | *TEL* | *INT* | *RFV* | *HCL* | *OOW* | *SCL* |
| --- | --- | --- | --- | --- | --- | --- | --- | --- | --- |
| *Variance Inflation Factor* | *VIF* | *2.94* | *1.17* | *1.70* | *1.27* | *1.01* | *1.92* | *1.55* | *1.04* |
|  | *1/VIF* | *0.3398* | *0.8536* | *0.5867* | *0.7876* | *0.9881* | *0.5211* | *0.6452* | *0.9572* |
| *correlation coefficient* | *NEU* | *1* |  |  |  |  |  |  |  |
|  | *FDI* | *0.2341* | *1* |  |  |  |  |  |  |
|  | *TEL* | *0.5943* | *0.3153* | *1* |  |  |  |  |  |
|  | *INT* | *0.4203* | *-0.0321* | *0.3090* | *1* |  |  |  |  |
|  | *RFV* | *0.0176* | *-0.0254* | *-0.0221* | *0.0225* | *1* |  |  |  |
|  | *HCL* | *0.6714* | *0.2274* | *0.4117* | *0.3228* | *0.0117* | *1* |  |  |
|  | *OOW* | *0.5489* | *0.2205* | *0.4527* | *0.2021* | *-0.0250* | *0.2783* | *1* |  |
|  | *SCL* | *0.0850* | *-0.0203* | *0.0344* | *0.0772* | *-0.0814* | *0.1467* | *-0.0531* | *1* |

(3) Data preprocessing. To improve the clarity of the methodology, we have described the data preprocessing steps taken prior to the analysis at the end of the first paragraph of the “3.3. Data sources and descriptive statistics” section. The specific details are as follows:

*A small number of missing values in NEU, FDI and ENC are complemented by linear interpolation. In order to prevent extreme values from interfering with the accuracy of the regression results, the data of GIN and GNS has undergone winsorization at the 1% level to eliminate the possible abnormal fluctuations in the data. The results of the descriptive statistical analysis are shown in Table 4. In order to make further empirical analysis, the main independent variable NEU and the dependent variables GIN and GNS are standardized. The standardized coefficients can directly compare the influence degree of variables, so as to improve the interpretability of the model results.*

(4) In the “ii) Independent variable” section of “3.1. Indicator design”, we have completed the description of the calculation process for the entropy weight method.

**Comment 6:**

6.Empirical Analysis

•Interpret coefficients in terms of real-world economic magnitude.

•Consider applying spatial econometric or dynamic panel models.

**Response:**

Thank you for your suggestions on the empirical analysis. We have made the corresponding modifications to the regression coefficients and the dynamic panel model. The specific changes are as follows:

(1) We have provided detailed explanations of the regression coefficients in “4.1 Benchmark regression” and “4.4 Moderating effects,” and illustrated the actual magnitude of the effect of NEU using real economic units. For example, in “4.1 Benchmark regression,” we analyzed that the coefficients of NEU on GIN and GNS are 0.6499 (p < 0.01) and 0.6744 (p < 0.01), meaning that for each one-unit increase in NEU, GIN increases by an average of 0.6499 units and GNS increases by an average of 0.6744 units. The adjusted R² values increased from 0.462 to 0.868 in Models E1 and E3, and from 0.344 to 0.858 in Models E2 and E4, indicating an improvement in overall model fit. In terms of real economic significance, these coefficients demonstrate that the "quality improvement" effect of NEU on GI is stronger than the "quantity increase" effect of NEU on GI, and NEU in China is in a transitional phase from "quantity-based growth" to "quality-oriented enhancement". This shows that China is shifting from the traditional “scale-driven” growth model to an “innovation-driven” development model in the new era. NEU is no longer simply a matter of population migration; it is now actively exerting the effects of factor agglomeration and high-quality innovation, guiding industries toward green, low-carbon, and modernized transformation. This also confirms the views of endogenous growth theory and innovation theory, namely, that the spatial agglomeration of economic activities effectively promotes innovation. The industrial upgrading and environmental regulations brought about by NEU compel firms to proactively seek fundamental change and engage in GI in order to gain competitive advantages. As a crucial component of this process, the “quality improvement” of GI better reflects the deep value of MEU than a mere “quantity increase.” NEU has solved the conditions of "consumer demand" and "talent supply" for quantity increase and quality improvement of GI. In addition, NEU advocates the green development mode and high-efficiency development mode, and the strict environmental standards force enterprises to improve efficiency and promote quantity increase and quality improvement of GI. At the critical stage of current social development, NEU is playing an increasingly important role in GI, and effectively promoting the rapid development and efficient application of GI in various fields.

For other coefficients, we have also provided detailed interpretations from a real economic perspective. The specific modifications have been marked in the manuscript.

(2) First and foremost, we sincerely thank you for your valuable suggestions on our paper. You suggested considering the application of spatial econometrics or dynamic panel models, which is an extremely forward-looking and professional recommendation. It has made us deeply aware of the room for further optimization and expansion in our research methodology, and we fully agree with your viewpoint. Building upon the baseline two-way fixed effects model, this paper further constructs a dynamic panel data model to control for the temporal dynamic dependence of the dependent variables (GIN and GNS). We conduct regression analysis using a second-order lagged dynamic two-way fixed effects model. The main reasons for this consideration are as follows: First, the Generalized Method of Moments (GMM) is more suitable for panel data with a large number of individuals (N) but a relatively short time dimension (T). Our research sample consists of panel data from 284 cities over 12 years. When T is relatively large, GMM estimation generates a large number of lagged instrumental variables, which can lead to serious over-identification problems due to excessive lag orders and instrument proliferation, resulting in biased estimation outcomes. In our preliminary regressions, both the Sargan test and Hansen test were significant, indicating insufficient validity of the instrumental variables and lack of robustness in the GMM estimation results. Second, in dynamic panels with a longer time dimension, fixed effects estimation (FE) actually exhibits better consistency and reliability. It can effectively control for city-level individual heterogeneity and time trends, thereby avoiding the endogeneity issues associated with instrumental variable selection in GMM. Third, considering that GIN and GNS display strong long-term inertia and historical accumulation characteristics, to mitigate potential persistence and path dependence in the dependent variables, this study introduces the second-order lagged terms of the dependent variables—L2.GIN and L2.GNS—on the basis of the baseline two-way fixed effects model, and constructs a dynamic panel model for robustness testing.

Based on the results of the dynamic two-way fixed effects regression, we have added a new subsection vi) Dynamic two-way fixed effects analysis in the “4.2 Robustness test” section. The specific content is as follows, and the regression results have been included in Table 8:

*To prevent the serious over-identification problem caused by too many lag orders and excessive expansion of instrumental variables in GMM analysis, and to alleviate the long-term persistence and long-term path dependence problems that may exist in dependent variables, this paper refers to the existing literature. Based on the benchmark two-way fixed effect model, the second-order lag term of the dependent variable (L2.GIN and L2.GNS) is introduced to construct a dynamic panel model for robustness testing [60,61]. Moreover, the second-order lag can capture the long-term dynamic characteristics more fully than the first-order lag. The regression results (in Table 8) show that the coefficients of the second-order lag term L2. GIN and L2. GNS are 0.4723 and 0.4318, respectively, which are significantly positive, indicating that GIN and GNS have a significant long-term path dependence effect, and the historical state has a continuous and stable positive impact on the current GI. After controlling the long-term dynamic inertia, cities fixed effect and years fixed effect, the coefficients of NEU to GIN and GNS are 0.0792 and 0.1486, respectively, which are significant, and the coefficient direction is consistent with the benchmark regression. It shows that the dynamic double fixed effect model not only does not negate the conclusion of the benchmark regression, but further supports the robustness of the results. Therefore, this paper still uses the static two-way fixed effect model as the core conclusion, and the dynamic model is only a robustness supplement.*

**Comment 7：**

7.Further Analysis

•Provide theoretical interpretation of the mechanisms, rather than relying solely on statistical significance.

**Response:**

We fully agree that relying solely on statistically significant results is not sufficient to fully demonstrate the threshold role of government environmental protection budget support in the impact of NEU on GI. To address this, we have systematically elaborated on the formation mechanism of the threshold effect by drawing on existing literature and theoretical models. In the “5 Further analysis” section, we have added a mechanism analysis subsection to strengthen the explanatory power. The specific content is as follows:

*From the perspective of factor supply, when the government 's support for green industries is low, green R&D projects will be limited by the support of funds and policies [74]. As the government 's support for green industries gradually in-creases, the financial constraints of GI are alleviated. When NEU needs a lot of green infrastructure and technology, financial support just makes up for the shortage of funds, making it easier for the urbanization process to drive the “quantity increase” of GI. Moreover, the government 's support behavior will also play a guiding role in green development and inhibit pollution, which will help limit high-emission and high-energy-consuming industries, and promote new formats such as pollution monitoring equipment and solid waste resource utilization [75]. Therefore, as the government 's green support increases, it helps facilitate NEU's "quantity increase" effect on GI.*

*The “quality improvement” effect of GI often requires policy support and continuous investment. When the GES is low, the investment in green R & D depends on the enterprise itself, which affects the continuous investment in R & D, and the GI “quality improvement” is limited. When the GES reaches a high level, government funds can support enterprises to carry out long-term technology iteration and result verification, so that enterprises are willing to invest more time and resources in "quality improvement" to optimize the stock technology and transform the process flow, and lead it to high efficiency and green transformation [76]，so as to improve the technical level and practical value, and promote more high-quality GI results. Moreover, from the perspective of technology diffusion, when GES is high, enterprises can participate in cross-regional and cross-industry exchanges and cooperation, which is more conducive to promoting technological exchanges to achieve deep knowledge integration and re-innovation[77]，promote green technology from follow-up innovation to leading innovation, and the quality of innovation results has been significantly improved. Therefore, with the increase of government green support, it is beneficial to expand the “quality improvement” effect of NEU on urban GI.*

**Comment 8：**

8.Discussion

•Deepen the theoretical interpretation of the findings.

•Compare the results with international studies on urbanization and innovation.

•Add a clear subsection on limitations and directions for future research.

**Response:**

Your comments are of vital importance for enhancing the theoretical depth of this study. In the revised manuscript, we have made the following improvements:

(1) In the original study, although we provided some theoretical elaboration of the findings, there was indeed a lack of depth and comprehensiveness. We first summarized and synthesized the main conclusions of this study, then re-examined and analyzed the theoretical implications of these conclusions. By further exploring the theoretical significance of the research, we integrated our findings with mainstream contemporary urban economic theory, green innovation studies, the Porter Hypothesis, endogenous growth theory, and the distinctive characteristics of the current “period of economic transition,” clarifying the intrinsic logical connections between this study and these theories, and highlighting the theoretical contributions and value of our work.

We have added the following specific content to the “6.2. Theoretical and policy implications” section:

*Our research focuses on the impact of NEU on urban GI, challenging the tradi-tional view that urbanization focuses on economic growth and resource utiliza-tion. NEU emphasizes green development and ecological benefits, and its role in promoting GI is more obvious, which is in line with the new urban development theory. It provides a new theoretical perspective for how urbanization promotes GI and enriches the existing theory of the relationship between urbanization and innovation. This paper discusses the “quantity increase” and “quality improvement” effect of NEU on GI, expands the theoretical framework of GI research from one-dimensional to two-dimensional, and enriches the research results of NEU. DIF amplifies the effect of NEU on urban GI, revealing its nonlinear characteristics and multivariate synergistic regulation mechanism (digital inclusive finance, government technology support, informatization, re-gional economy, energy consumption). This is not only the deepening of Porter hypothesis and endogenous growth theory in the context of green transformation, but also helps to reveal the internal logic and complex mechanism of NEU affecting urban GI more comprehensively and deeply. It also provides a new theoretical explanation dimension for understanding how the institutional, financial and technological environment jointly shape the impact path of urban-ization on innovation. This study not only provides policy reference for the construction of NEU, but also provides important empirical evidence and new analytical perspective for the development of urbanization-GI theory with the characteristics of “transitional economy”, and provides new evidence for veri-fying and expanding international research on the interaction mechanism of ur-banization, digital finance and GI.*

(2) We have extensively reviewed the latest international literature on urbanization and green innovation, situating our research findings within the global research framework on urbanization and green innovation for comparative analysis. Through this comparison with international studies, we have clarified the positioning and contributions of our research, highlighted similarities with existing studies, and reaffirmed the unique value of our work. We have also identified the limitations of our own study and the gaps between our findings and the current frontiers of academic research, which points the way for our future research and scholarly exploration.

We have added the following content at the end of the first paragraph of the “6.2. Theoretical and policy implications” section:

*Compared with the relevant international research, Existing research mainly analyzes that NEU can promote urban GI from the perspective of enterprises [78], policy [79,80] and green development [81]. However, the urbanization models in different regions are different, and the impact of different urbanization models on regional green technology R&D are also different [8]. Moreover, the impact of NEU on GI also needs to be played through mediating variables. For example, NEU promotes GI by alleviating corporate financial constraints [9]. In addition, existing international research often considers NEU as an independent driving factor into the innovation system for research. This study not only analyzes the “quantity increase” and “quality improvement” effects of NEU and urban GI, but also incorporates NEU with DIF, GTS, INT, AGDP, and ENC into the same analytical framework, revealing the complex mechanism, which provides a new perspective for understanding the relationship between NEU and GI in different contexts.*

(3) In the revised manuscript, we have added a separate section to discuss the limitations of this study and directions for future research. Regarding limitations, we conducted an objective analysis focusing on aspects such as indicator measurement, research methodology, and the applicability of the research conclusions. In response to these limitations, we put forward corresponding improvement measures and suggestions based on current research hotspots and practical needs, hoping to contribute to the further development of this field.

We have supplemented the following content to the “6.3. Limitations and future research” section:

*It should be pointed out that while we use green patent applications and green invention patent applications as measures for the "quantity increase" of GI and "quality improvement" of GI, respectively, relying solely on patent data as a proxy for all GI has several potential limitations. Because not all innovations are patented, it is challenging to capture the full scope of GI using patent data. In addition, although we have employed various methods in the paper to test the robustness and endogeneity of the analysis results, in order to alleviate concerns about reliability on specific data proxies, data and methods limitations still exist. China has a distinctive social system and urbanization process, and this study is mainly based on the analysis of China's urban panel data, which will inevitably affect the applicability of the research conclusions to other countries.*

*To further overcome these limitations, patent data can be combined with other data in future research on GI, and environmental performance indicators, energy efficiency, etc., can be included in the analysis of GI to more compre-hensively cover the scope of GI. In terms of research methods, future research needs to use spatial econometric analysis methods or more rigorous causal in-ference methods to further verify the causal relationship between new urbaniza-tion and green innovation，such as incorporating qualitative research methods or adopting quasi-experimental research methods, to provide more causal evidence for the relationship between NEU and GI. Future research also can further ex-pand the scope of the study, analyze the relationship between urbanization and GI in other countries, and compare it with China to test the boundary conditions of our research results.*

**Comment 9：**

9.References

•Ensure consistency in DOI formatting.

•Confirm that all cited references appear in the reference list and that all listed references are cited in the text.

•Prioritize the most recent and relevant literature-preferably published between 2017 and 2026-to maintain the currency of the review. Where appropriate, removing outdated methodological references may further strengthen the manuscript.

**Response:**

In response to your revision suggestions, we have addressed each point and completed the corresponding amendments.

First, we thoroughly checked the DOI annotation format for all entries in the reference list and uniformly adjusted them to the international standard format (i.e., https://doi.org/10.xxxx/xxxxxx).

Second, we verified that every citation in the main text corresponds to a complete entry in the reference list. We examined all items in the reference list to ensure that each source is explicitly cited in the text, with no redundant or missing references. At present, the references and citations are fully matched.

Finally, we searched for and added core journal articles published between 2022 and 2026, replacing some earlier citations. For the retained literature, we assessed the relevance of each study’s topic to this paper to ensure that every reference effectively supports the core arguments of our work.

**Once again, thank you for your professional guidance. We will continue to refine and improve the quality of our manuscript.**

***Response to Reviewer #8 Comments***

**Reviewer #8:**

The Impact of new urbanization on quantity increase and quality improvement of green innovation: Based on multivariate moderating effect models, Manuscript Number: PONE-D-25-37471R2

General issues (taking into account seven guiding principles): Since I have viewed both the original and revised paper.

**Response:**

We are writing to express our deepest gratitude for your encouraging review of our manuscript (PONE-D-25-37471R2). We are truly honored by your positive assessment across all seven guiding principles of PLOS ONE and your recommendation for publication. Your recognition is a significant source of motivation for us.

We have carefully read all of your comments and are pleased to confirm that the manuscript has been prepared in accordance with the high standards you have outlined. Please find our point-by-point response below:

**Comment 1:**

A1. The research is the original results of the study. The paper explores the linkage between new urbanisation and green innovation practices in Chinese cities, hence, presenting an example that can be used in policy and research in other nations and in filling the research gap in the field.

**Response:**

We are pleased that the novel focus on the dual impact (quantity and quality) of green innovation within the context of new urbanization in China is seen as a valuable contribution to the global literature.

**Comment 2:**

A2. There has been no publication of the results obtained. The manuscript utilises a micro-panel dataset spanning 2011 to 2022 (11 years) that makes the authors depict the time-related tendency in green innovation in the context of new urbanisation.

**Response:**

We confirm that the results presented in this manuscript are entirely original and have not been published elsewhere. We have ensured that the temporal analysis is clearly presented in the revised text to highlight these trends.

**Comment 3:**

A3. Experiments, statistics, and other discussions are done to a high technical standard, and they are described in adequate detail. Basic pre-tests have been met, and the obtained estimates are sound, which makes it easy to replicate other researchers.

**Response:**

Thank you for confirming the technical rigor of our methodology. We have double-checked the statistical methods section to ensure that the description of the multivariate moderating effect models is sufficiently detailed to allow for replication by other scholars.

**Comment 4:**

A4. The conclusions (summary) are made in a suitable way and are backed up by data. The conclusions are made logically out of the key findings and presented in a clear, scientific and concrete way.

**Response:**

We have reviewed the conclusion section to ensure that every claim is directly supported by the empirical evidence derived from our analysis.

**Comment 5:**

A5. The article is written in the regular English. The paper is logically articulated and its structure is very well detailed.

**Response:**

We are very grateful for the reviewer’s positive assessment of our manuscript’s language and structure. To further enhance the quality of our work, we continue to strengthen the refinement of the language in our future revisions and submissions, striving to make the academic expression even more standardized, precise, and eloquent.

**Comment 6:**

A6. The study adheres to the necessary requirements regarding the ethics of research and experimentation. Following the examination of the document, critical remarks by reviewers and revision comments by authors were dealt with accordingly with the technical problems being fixed.

**Response:**

We confirm our adherence to all ethical requirements. We have diligently addressed all previous critiques, and we view the constructive feedback from reviewers as an essential part of the scholarly process that helps us grow as researchers.

**Comment 7:**

A7. The article follows the right reporting conventions and research ethics concerning data publication.

Hence, I would advise that the paper can be published in PLOS ONE journal.

**Response:**

We have verified that the manuscript strictly follows the appropriate reporting guidelines and that the data handling process meets the journal's ethical requirements.

**Once again, thank you for your professional guidance. We will continue to improve the quality of the manuscript.**
